# National Assessment of Statin Therapy in Patients Hospitalized with Acute Myocardial Infarction: Insight from China PEACE-Retrospective AMI Study, 2001, 2006, 2011

**DOI:** 10.1371/journal.pone.0150806

**Published:** 2016-04-08

**Authors:** Lihua Zhang, Jing Li, Xi Li, Khurram Nasir, Haibo Zhang, Yongjian Wu, Shuang Hu, Qing Wang, Nicholas S. Downing, Nihar R. Desai, Frederick A. Masoudi, John A. Spertus, Harlan M. Krumholz, Lixin Jiang, China PEACE Collaborative Group

**Affiliations:** 1 National Clinical Research Center of Cardiovascular Diseases, State Key Laboratory of Cardiovascular Disease, Fuwai Hospital, National Center for Cardiovascular Diseases, Chinese Academy of Medical Sciences and Peking Union Medical College, Beijing, People's Republic of China; 2 Center for Healthcare Advancement & Outcomes, Baptist Health South Florida, Miami, Florida, United States of America; 3 Miami Cardiac & Vascular Institute, Baptist Health South Florida, Miami, Florida, United States of America; 4 The Center for Outcomes Research and Evaluation, Yale-New Haven Hospital, New Haven, Connecticut, United States of America; 5 Division of Cardiology, University of Colorado Anschutz Medical Campus, Aurora, Colorado, United States of America; 6 Saint Luke’s Mid America Heart Institute, University of Missouri Kansas City, Kansas City, Missouri, United States of America; 7 Section of Cardiovascular Medicine and the Robert Wood Johnson Clinical Scholars Program, Department of Internal Medicine, Yale University School of Medicine, New Haven, United States of America; 8 Department of Health Policy and Management, Yale School of Public Health, New Haven, United States of America; Nagoya University, JAPAN

## Abstract

**Background:**

Statin therapy is among the most effective treatments to improve short- and long-term mortality after acute myocardial infarction. The use of statin, and the intensity of their use, has not been described in acute myocardial infarction patients in China, a country with a rapidly growing burden of cardiovascular disease.

**Methods and Results:**

Using a nationally representative sample of patients with acute myocardial infarction admitted to 162 Chinese hospitals in 2001, 2006 and 2011, we identified 14,958 patients eligible for statin therapy to determine rates of statin use and the intensity of statin therapy, defined as those statin regimens with expected low-density lipoprotein cholesterol lowering of at least 40%, to identify factors associated with the use of statin therapy. Statin use among hospitalized patients with acute myocardial infarction increased from 27.9% in 2001 to 72.5% in 2006, and 88.8% in 2011 (*P*<0.001 for trend). Regional variation in statin use correspondingly decreased over time. Among treated patients, those receiving intensive statin therapy increased from 1.0% in 2001 to 24.2% in 2006 to 57.2% in 2011(*P*<0.001 for trend). Patients without low-density lipoprotein cholesterol measured were less likely to be treated with statin or to receive intensive therapy.

**Conclusions:**

The use of statin therapy has dramatically increased over the past decade in Chinese patients with acute myocardial infarction. However, half of patients still did not receive intensive statin therapy in 2011.Given that guidelines strongly endorse intensive statin therapy for acute myocardial infarction patients, initiatives promoting the use of statin therapy, with attention to treatment intensity, would support further improvements in practice.

## Introduction

China, a country of more than 1.3 billion people, faces a marked increase in the incidence of acute myocardial infarction (AMI) with an estimated 23 million patients forecasted to experience AMI in 2030[1].This creates an imperative to optimize the use of evidence-based therapies that improve patient outcomes. There is compelling scientific evidence demonstrating that statin therapy reduces the rate of major adverse cardiovascular events in patients following after AMI, with so-called “intensive” statin regimens offering the greatest benefit [2–7].

Clinical guidelines around the world strongly endorse statin therapy for all patients with AMI [8,9]. More recently, guidelines endorsed by the American Heart Association and American College of Cardiology (AHA/ACC), recommended “high intensity” statin therapy (defined as those regimens that lower low-density lipoprotein cholesterol (LDL-C) by at least 50%) for patients with AMI [10]. The most recent Chinese guidelines for both non-ST elevation myocardial infarction (published in 2007) and ST elevation myocardial infarction (published in 2010) recommend statin therapy in all patients with AMI, but do not provide specific guidance about the intensity of such therapy [11,12]. However, the Chinese dyslipidemia guidelines (published in 2007) recommend “intensive” statin treatment in all patients with AMI regardless of baseline LDL-C [13]. Notably, Chinese guidelines define intensive statin therapy as any statin regimen that lowers LDL-C by at least 40% (as opposed to the 50% reduction required by the ACC/AHA guidelines). This definition is congruent with observations that Chinese patients, as compared with Caucasian patients, have lower LDL-C levels, and are more likely to experience adverse reactions to statins[14].

A recent analysis of a nationally-representative sample of Chinese patients with STEMI, statin use increased 3-fold between 2001 and 2011[15].Although this study provided initial insights into the extent of statin use in China, it did not provide information about use in a broad spectrum of patients with AMI, the intensity of statin therapy, or regional variation in use. Given that health care resources are not evenly distributed in China [16], it is important to understand if there are disparities in the use of statins among patients with AMI in China. Finally, understanding factors associated with lack of statin use can inform efforts to improve current prescription practices of statin therapy.

Accordingly, we performed a detailed analysis of statin utilization as part of a national quality assessment of AMI that uses data collected in the China Patient-Centered Evaluative Assessment of Cardiac Events Retrospective Study of Acute Myocardial Infarction (China PEACE-Retrospective AMI Study).Specifically, our research objectives were to:(1) determine current rates of and temporal trends in statin therapy, and intensive statin therapy, among eligible patients hospitalized with AMI in China; (2) assess regional variation in statin therapy; (3) examine the use of statin therapy by LDL-C level; (4) analyze factors associated with the lack of any statin therapy and intensive statin therapy in 2011.

## Methods

### Design Overview of China PEACE-Retrospective AMI Study

**Fig 1:**
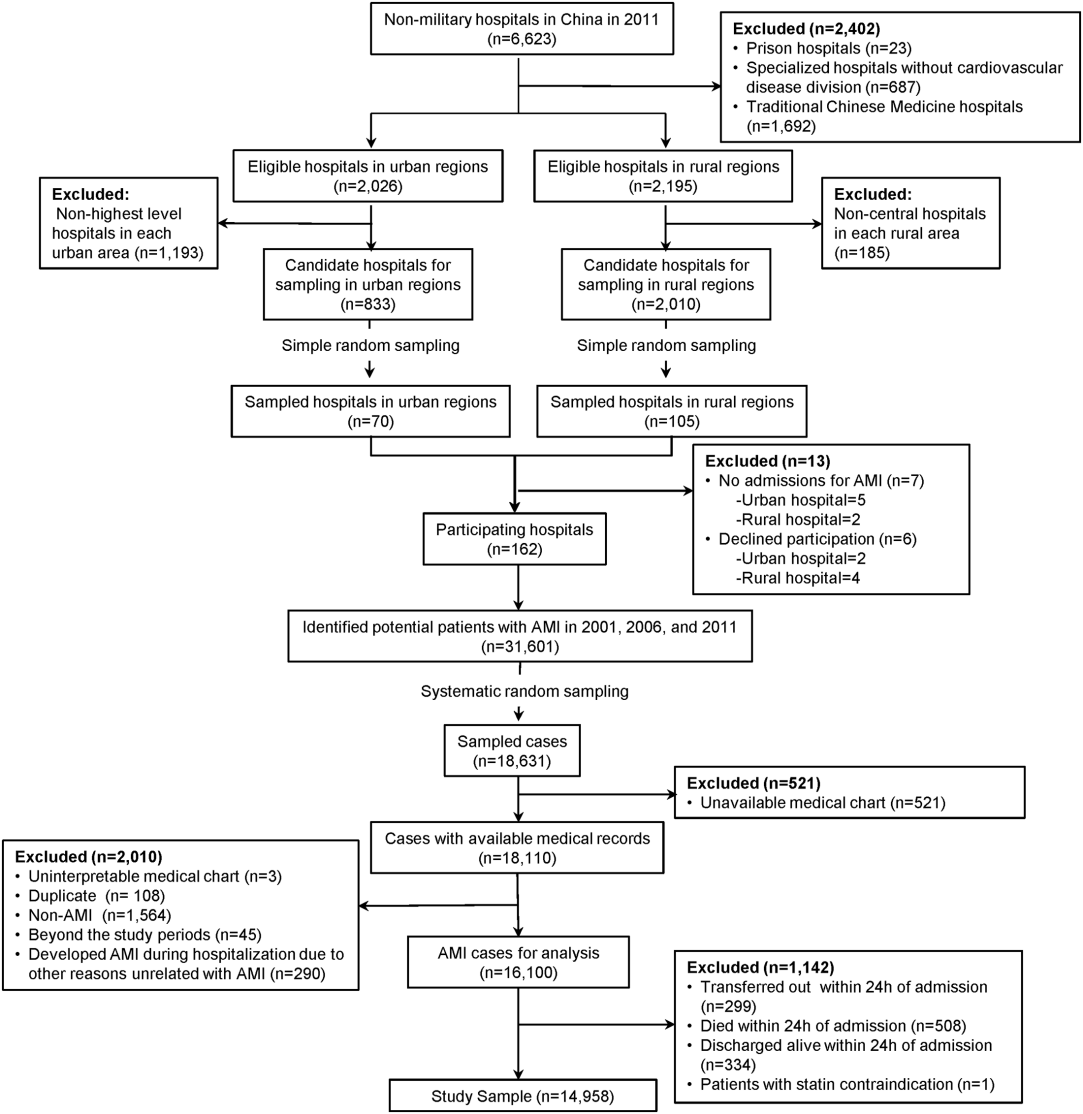
Flow Diagram of Study Sample.

Full details of the design and methods of the China-PEACE Retrospective-AMI study have been published previously [17]. In brief, we created a nationally representative sample of AMI hospitalizations during 2001, 2006, and 2011 with a two-stage random sampling design. In the first stage, we identified hospitals using a simple random sampling procedure within each of the 5 study strata: Eastern-rural, Central-rural, Western-rural, Eastern-urban, and Central/Western-urban regions. Since hospital volumes and clinical capacities differ between urban and rural areas as well among the three official economic-geographic regions (Eastern, Central, and Western) of Mainland China, we combined Central and Western urban regions together given their similar per capita income and health services capacity. According to government documents, there were 6623 non-military hospitals in 2011. We excluded prison hospitals, specialized hospitals without a cardiovascular disease division, and traditional Chinese medicine hospitals. In the 3 rural strata, the sampling framework consisted of the central hospital in each of the predefined rural regions (2010 central hospitals in 2010 rural regions). In each of the 2 urban strata, the sampling framework consisted of the highest-level hospitals in each of the predefined urban regions (833 hospitals in 287 urban regions) (Fig 1). We randomly sampled all the central hospitals in rural regions and all of the highest-level hospitals in urban regions, and excluded hospitals that neither admitted patients with acute myocardial infarction or refused to participate. Since the majority of hospitals in China are publicly owned and administered, hospital closure is rare. The study cohort should be most representative of national treatment patterns and outcomes in 2011. Therefore, we selected representative hospitals from 2011 to reflect current practices and traced this cohort of hospitals back to 2006 and 2001 to describe temporal trends. In the second sampling stage, we identified cases based on the local hospital database for patients with AMI in each year at each sampled hospital using systematic random sampling procedures. In each of the 5 study strata, we determined the sample size required to achieve a 2% precision for describing the primary outcome, in-hospital mortality, which we had estimated to be approximately 9% in urban hospitals and 7% in rural county-level hospitals. We doubled the cluster sizes for 2011 to improve precision in the description of contemporary hospital-level treatment patterns and outcomes. Patients with AMI were identified using International Classification of Diseases—Clinical Modification codes, including versions 9 (410.xx) and 10 (I21.xx)–information that the Ministry of Health in China requires to be included in medical charts. When this data was unavailable, site coordinators manually searched the hospital’s written log to identify hospitalizations for AMI. Only patients with a definite discharge of AMI were included in the study sample. In any case where the diagnosis of AMI was unclear, the site coordinator reviewed the original medical records.

Data were collected via standardized central medical record abstraction using standardized data definitions. We adopted rigorous monitoring at each stage to ensure data quality. Data abstraction quality was monitored by randomly auditing 5% of records, with overall accuracy of the variables being more than 98% [17].

The central ethics committee (Ethics Committee of Fuwai Cardiovascular Hospital) at the China National Center for Cardiovascular Diseases approved the China PEACE-Retrospective AMI Study. All collaborating hospitals accepted the central ethics approval except for five hospitals, which obtained local approval by internal ethics committees (Ethics Committee of Jilin Province People's Hospital, Ethics Committee of Tianjin Medical University General Hospital, Ethics Committee of HuaXin Hospital, First Hospital of Tsinghua University, Ethics Committee of Affiliated Zhongshan Hospital of Dalian University, Ethics Committee of The First People's Hospital of Guangyuan).Consent form was not required because we got data from retrospective medical chart review. Participating hospitals copied and transmitted the records to the NCCD after de-identification. The study is registered at www.clinicaltrials.gov (NCT01624883).

### Study Sample

We sampled 175 hospitals, of which 7 were excluded because they did not admit patients with AMI and 6 declined to participate. Within the 162 remaining hospitals, there were 31,601 hospitalizations for AMI in 2001, 2006 and 2011, from which we sampled 18,631 cases and acquired medical records for 18,110 cases (97.2%). After exclusion of cases not appropriate for study (including uninterpretable medical charts, duplicate cases, non-AMI diagnoses, cases not within study periods, and AMI that occurred during hospitalization), we identified 16,100 patients with a definitive discharge diagnosis of AMI that were representative of all patients hospitalized for AMI in China in 2001, 2006, and 2011. We excluded the following patients: who died within 24 hours (n = 508), were transferred out within 24 hours (n = 299), were discharged within 24 hours (n = 334), because these patients may not have the opportunity to receive statin therapy. Among the remaining patients, we excluded patients who had a documented contraindication to statin that was described by the physician in the medical record (n = 1), 14,958 patients with AMI, who were eligible for statin therapy remained and were included in our analysis (Fig 1): 2198 (14.7%) were hospitalized for AMI in 2001, 4159 (27.8%) in 2006 and 8601 (57.5%) in 2011.

### Variables

We abstracted information about patient demographics (age, gender), cardiovascular risk factors (hypertension, diabetes, dyslipidemia, smoking), medical history (myocardial infarction, ischemic stroke, hemorrhagic stroke), presenting symptoms(chest pain, cardiac arrest, cardiac shock), laboratory results, diagnostic procedures, treatments, timing of care delivery and hospital outcomes from the medical record; the full details of the medical record abstraction have been described previously [17]. The presence of comorbidities, including hypertension, diabetes, and dyslipidemia, were recorded when such conditions were documented by the treating physicians in the hospital medical record (admission notes, discharge diagnosis or positive laboratory test results). To ensure that all patients with dyslipidemia were identified, we further assigned a diagnosis of dyslipidemia on the basis of lipid panels measured during AMI admission. Dyslipidemia was defined as total cholesterol greater than 5.18 mmol/L,LDL-C greater than or equal to 3.37 mmol/L, or high-density lipoprotein less than 1.04 mmol/Lfor men or less than 1.30 mmol/L for women [18].

### Statin Therapy and Intensive Statin Therapy

The type and dosage of statin therapy were determined by reviewing the last physician order before discharge or in-hospital death. Subsequently, we identified patients receiving intensive statin therapy, which was defined as statins dosed at a level expected to lower LDL-C by at least 40% according to the Chinese guideline[13], rather than the ACC/AHA recommendation for doses expected to lower LDL-C by at least 50% [10]. This difference reflects the lower average LDL-C levels of Chinese and the concern about safety [14]. The details of statin regimens grouped by intensity were shown in (S1 Table) [10,19].

### Statistical analysis

Categorical variables were described as frequencies with percentages. We transformed two continuous variables (i.e. age and LDL-C level) into categorical variables according to clinically meaningful cutoff values. We categorized age by 10-year age groups (<55, 55–64, 65–74, ≥75 years) and LDL-C levels by clinically meaningful cutoff values (<1.81, 1.81–2.59, 2.60–3.37, >3.37 mmol/L). Missing age values, which occurred infrequently (0.1%), were imputed as the overall median. To assess differences between patients with and without statin therapy, we used chi-square testing for categorical variables.

To estimate the use of statin therapy among the entire Chinese population with AMI, we applied weights proportional to the inverse sampling fraction of hospitals within each stratum, and the sampling fraction of patients within each hospital to account for differences in the sampling fraction for each time period. We also stratified the unweighted rate of statin therapy among eligible patients by the five defined regions and baseline LDL-C level, separately. Trends in the use of statin therapy over time were evaluated by Cochran-Armitage test for trend.

To adjust for multiple factors and identify factors associated with any statin therapy, a multilevel logistic regression model using generalized estimating equation to account for clustering of patients within hospitals was used. In order to reflect current practice patterns, we restricted this analysis to patients hospitalized in 2011.Candidate explanatory variables, which were based on clinical judgment and literature review, included demographic characteristics (age and gender), cardiovascular risk factors (hypertension, diabetes, dyslipidemia, smoker) medical history (myocardial infarction, ischemic stroke, hemorrhagic stroke), clinical characteristics at admission (chest discomfort, cardiac arrest, cardiac shock), in-hospital management(fibrinolysis, percutaneous coronary intervention), AMI type (STEMI, NSTEMI), LDL-C level, economic-geographic region characteristics and urban/rural region. Similarly, we examined patient characteristics associated with receiving intensity statin therapy among patients who were hospitalized in 2011 and had documented statin dosage. We included all above candidate variables. We performed backward stepwise selection method (with a cutoff significance level of 0.05) to determine variables for inclusion in the final model. Odds ratios (OR) and 95% confidential intervals (CI) were reported for both logistic regression analyses. All tests of statistical significance were 2-sided, with a *P*<0.05 considered statistically significant. Statistical analysis was performed using SAS software (version 9.2, SAS Institute, Cary, NC).

## Results

### Study Sample

There were 14,958 patients with AMI in our study. The proportion of male patients was 70.3%. Comorbidities were common, 60.5% of patients had dyslipidemia, 50.9%hypertension, and 20.3% diabetes. Additionally, prior cardiovascular disease was frequent, with 10.8% having a history of myocardial infarction and 10.3% having history of ischemic stroke (Table 1).

**Table 1:** Baseline Characteristics of the Study Cohort Stratified by Statin Therapy.

Characteristics	Total NO (%)		Statin Therapy NO (%)	No Statin Therapy NO(%)	*P* value
All eligible patients	14,958		11,268(75.3)	3690(24.7)	<0.001
Demographic					
Age, years					0.092
<55	3346(22.4)		2563(22.7)	783(21.2)	
55–64	3562(23.8)		2710(24.1)	852(23.1)	
65–74	4471(29.8)		3258(28.9)	1213(32.9)	
≥75	3579(23.9)		2737(24.3)	842(22.8)	
Gender					
Female	4446(29.7)		3306(29.3)	1140(30.9)	0.073
CVD risk factors					
Prior hypertension	7430(49.7)		5908(52.4)	1522(41.2)	<0.001
Prior diabetes	2603(17.4)		2134(18.9)	469(12.7)	<0.001
Prior dyslipidemia	9050(60.5)		7410(65.8)	1640(44.4)	<0.001
Current smoker	5242(35.0)		4197(37.2)	1045(28.3)	<0.001
Medical histories					
Myocardial infarction	1615(10.8)		1267(11.2)	348(9.4)	0.002
Ischemic stroke	1534(10.3)		1159(10.3)	375(10.2)	0.831
Hemorrhagic stroke	184(1.2)		134(1.2)	50(1.4)	0.428
Clinical characteristics at admission					
Chest discomfort	13751(91.9)		10467(92.9)	3284(89.0)	<0.001
Cardiac arrest	176(1.2)		141(1.3)	35(0.9)	0.138
Cardiac shock	644(4.3)		462(4.1)	182(4.9)	0.031
AMI type					
STEMI	12806(85.6)		9569(84.9)	3237(87.7)	<0.001
Laboratory test					
LDL-C level, mmol/L					<0.001
< 1.81	1443(9.6)		1158(10.3)	285(7.7)	
1.81–2.59	3677(24.6)		3030(26.9)	647(17.5)	
2.60–3.37	3803(25.4)		3166(28.1)	637(17.3)	
>3.37	2707(18.1)		2291(20.3)	416(11.3)	
Unmeasured	3328(22.2)		1623(14.4)	1705(46.2)	
Economic-geographic region					<0.001
Eastern	8866(59.3)		6783(60.2)	2083(56.4)	
Central	3195(21.4)		2246(19.9)	949(25.7)	
Western	2897(19.4)		2239(19.9)	658(17.8)	
Urban/Rural					
Rural	5664(37.9)		3796(33.7)	1868(50.6)	<0.001
Urban	9294(62.1)		7472(66.3)	1822(49.4)	
Year					<0.001
2001	2198(14.7)		613(5.4)	1585(43.0)	
2006	4159(27.8)		3016(26.8)	1143(31.0)	
2011	8601(57.5)		7639(67.8)	962(26.1)	

AMI indicates acute myocardial infarction;

STEMI indicates ST-segment elevation myocardial infarction;

LDL-C indicates low density lipid cholesterol.

### Statin therapy and Intensive Statin Therapy

Notably, the proportion of eligible patients receiving any statin therapy increased over the past decade from 27.9% (weighted rate 29.4%) in 2001, to 72.5% (weighted rate 74.8%) in 2006, and then to 88.8% (weighted rate 90.7%) in 2011 (*P*<0.001 for trend).

**Fig 2:**
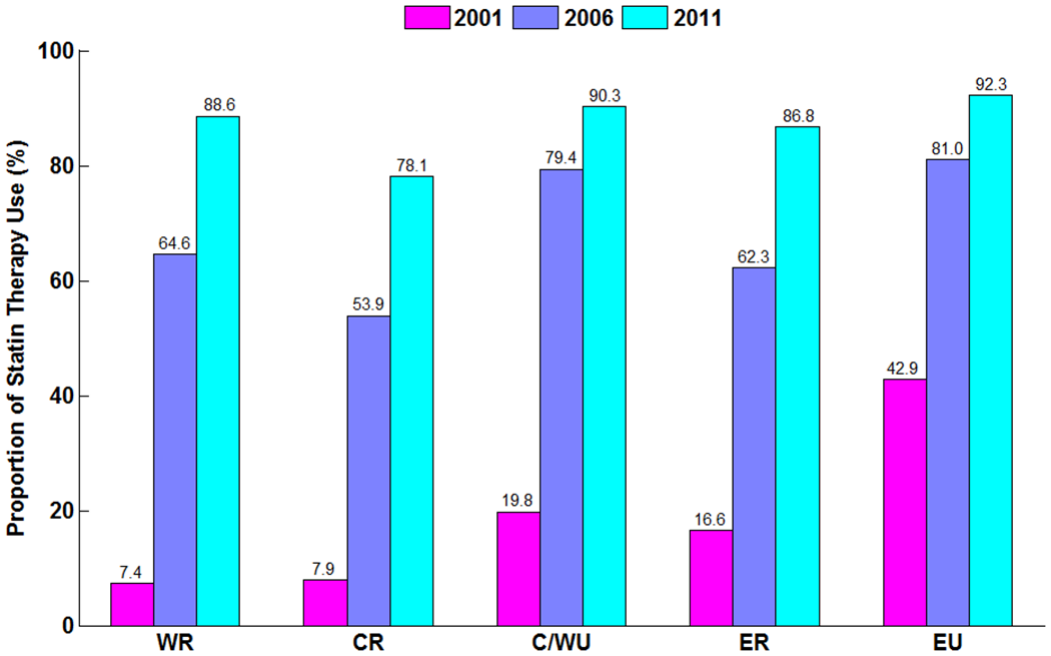
Proportion of Patients Receiving Statin therapy Stratified by Region. *P* for trend <0.001 for the proportion of statin therapy in 2001, 2006 and 2011.*P* for trend <0.001 for the proportion of statin therapy in different regions.WR indicates Western rural; CR indicates Central rural; ER indicates Eastern rural; C/WU indicates Central/Western urban; EU indicates Eastern urban.

The rate of statin therapy increased in all regions over time, and the variation in statin use across regions decreased. In 2001, the rate of statin use ranged from 7.4% in Western-rural to 42.9% in Eastern-urban, but by 2011, it ranged from 78.1% in Central-rural to 92.3% in Eastern-urban (Fig 2). Similarly, the rates of statin therapy increased overtime regardless LDL-C level (S1 Fig).

There were significant changes in the type of statin use during the past decade. Simvastatin was the most frequently used agent in 2001 (60.2%) and 2006(44.5%), but in 2011 atorvastatin became the principal statin, accounting for 52.9% of use (S2 Fig).

**Fig 3:**
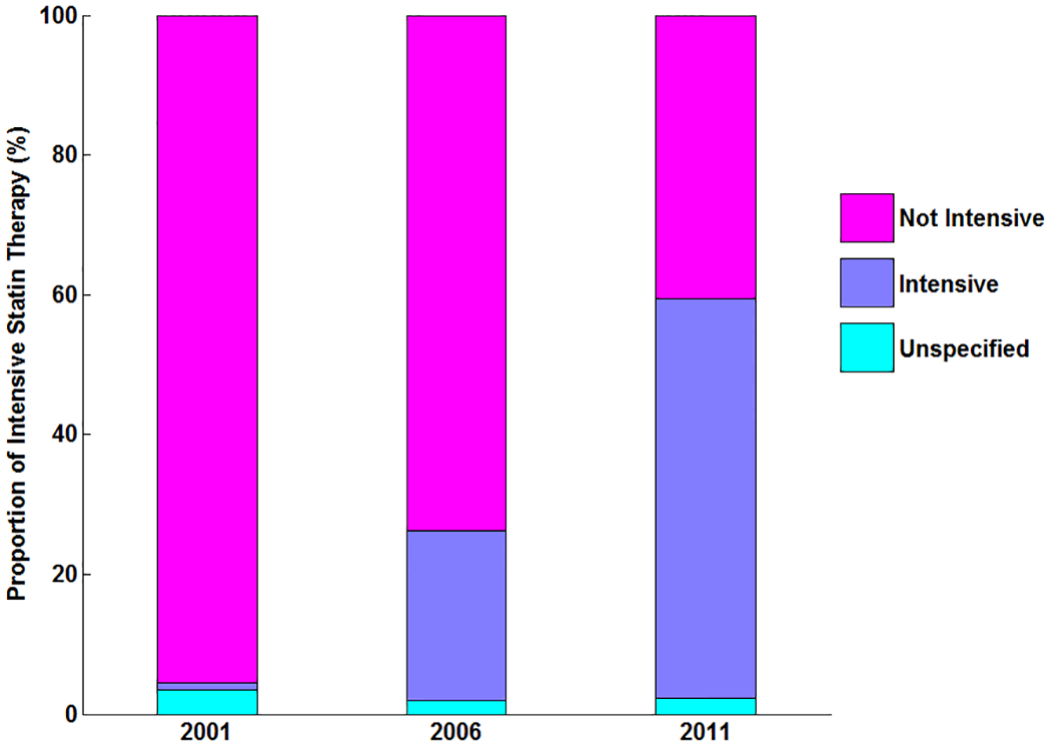
Statin Intensity Therapy among Patients with Statin Therapy in China. P for trend <0.001 for the proportion of intensive statin therapy in 2001, 2006 and 2011. WR indicates Western rural; CR indicates Central rural; ER indicates Eastern rural; C/WU indicates Central/Western urban; EU indicates Eastern urban.

**Fig 4:**
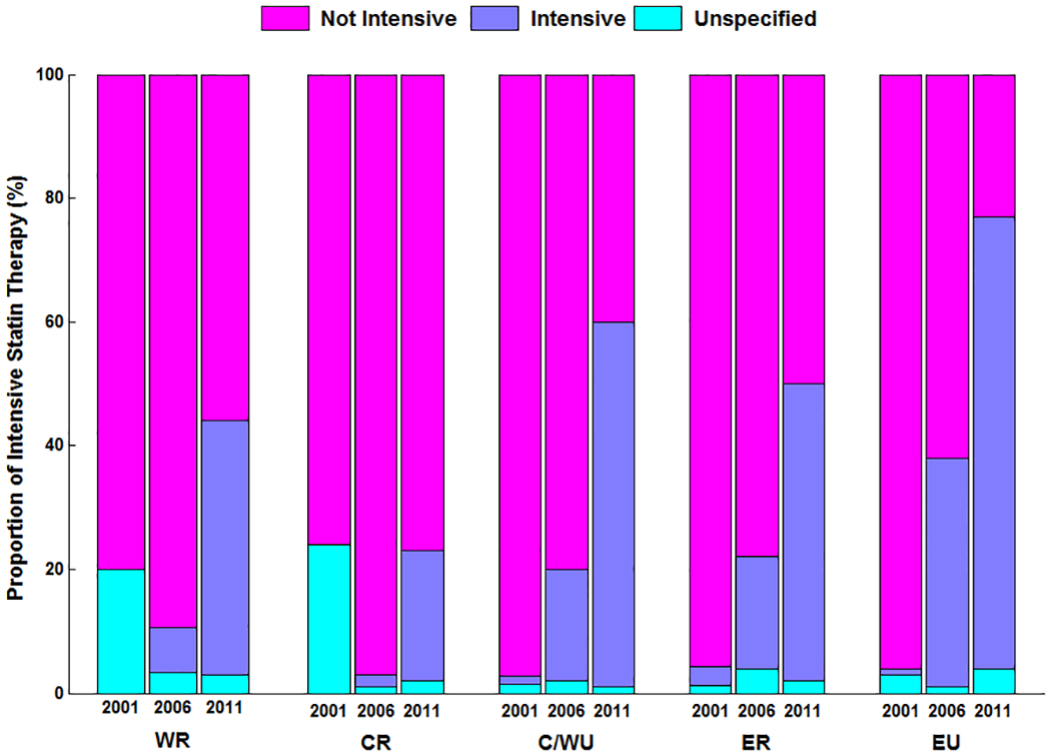
Statin Intensity Therapy among Patients with Statin Therapy Stratified by Region. P for trend <0.001 for the proportion of intensive statin therapy in different regions. WR indicates Western rural; CR indicates Central rural; ER indicates Eastern rural; C/WU indicates Central/Western urban; EU indicates Eastern urban.

Among patients treated with statins (Table 2), the proportion of patients receiving intensive statin therapy increased from1.0% in 2001 to 24.2% in 2006 and 57.2% in 2011(Fig 3). Although the rates of intensive statin therapy increased in all regions, wide variation persisted in 2011, ranging from 21.0% in the Central-rural region to 73.0% in Eastern-urban (P for overall<0.001) (Fig 4).

**Table 2:** Baseline Characteristics of Statin-Treated Patients Stratified by Treatment Intensity.

Characteristics	Total NO (%)	Intensive Statin Therapy NO (%)	Less Intensive Statin Therapy NO (%)	*P* value
All patients	11,010*	5106 (46.4)	5904 (53.6)	0.002
Demographic				
Age, years				
<55	2491(22.6)	1216(23.8)	1275(21.6)	0.826
55–64	2647(24.0)	1252(24.5)	1395(23.6)	
65–74	3177(28.8)	1400(27.4)	1777(30.1)	
≥75	2695(24.5)	1238(24.2)	1457(24.7)	
Gender				
Female	3230(29.3)	1378(26.9)	1852(31.4)	<0.001
CVD risk factors				
Prior hypertension	5921(53.8)	2848(55.8)	3073(52.0)	<0.001
Prior diabetes	2446(22.2)	1272(24.9)	1174(19.9)	<0.001
Prior dyslipidemia	7229(65.7)	3619(70.9)	3610(61.1)	<0.001
Current smoker	4118(37.4)	2041(39.9)	2077(35.2)	<0.001
Medical histories				
Myocardial infarction	1239(11.3)	604(11.8)	635(10.8)	0.075
Ischemic stroke	1136(10.3)	511(10.1)	625(10.6)	0.319
Hemorrhagic stroke	132(1.2)	61(1.2)	71(1.2)	0.969
Clinical characteristics at admission				
Chest pain	10224(92.9)	4757(93.4)	5457(92.4)	0.058
Cardiac arrest	137(1.2)	68(1.3)	69(1.2)	0.441
Cardiac shock	450(4.1)	216(4.2)	234(3.9)	0.481
AMI type				
STEMI	9344(84.9)	4223(82.7)	5121(86.7)	<0.001
Laboratory test				
LDL-C level, mmol/L				<0.001
< 1.81	1140(10.4)	529(10.4)	611(10.3)	
1.81–2.59	2952(26.8)	1405(27.5)	1547(26.2)	
2.60–3.37	3091(28.1)	1499(29.3)	1592(27.0)	
>3.37	2251(20.4)	1137(22.3)	1114(18.9)	
Unmeasured	1576(14.3)	536(10.5)	1040(17.6)	
Economic-geographic region				0.872
Eastern	6585(59.8)	3436(67.3)	3149(53.3)	
Central	2224(20.2)	843(16.5)	1381(23.4)	
Western	2201(20)	827(16.2)	1374(23.3)	
Urban/Rural				
Rural	3708(33.7)	1165(22.8)	2543(43.1)	<0.001
Urban	7302(66.3)	3941(77.2)	3361(56.9)	
Year				
2001	592(5.4)	6(0.1)	586(9.9)	<0.001
2006	2956(26.8)	729(14.3)	2227(37.7)	
2011	7462(67.8)	4371(85.6)	3091(52.4)	

*Note: 11,268 patients received statin therapy; however, the dose was not documented in the medical record of 258 patients and were excluded from this table, leaving11,010 patients whose statin dose was definitively recorded.

AMI indicates acute myocardial infarction;

STEMI indicates ST-segment elevation myocardial infarction;

LDL-C indicates low density lipid cholesterol.

### Factors associated with use of any statin therapy in 2011

**Fig 5:**
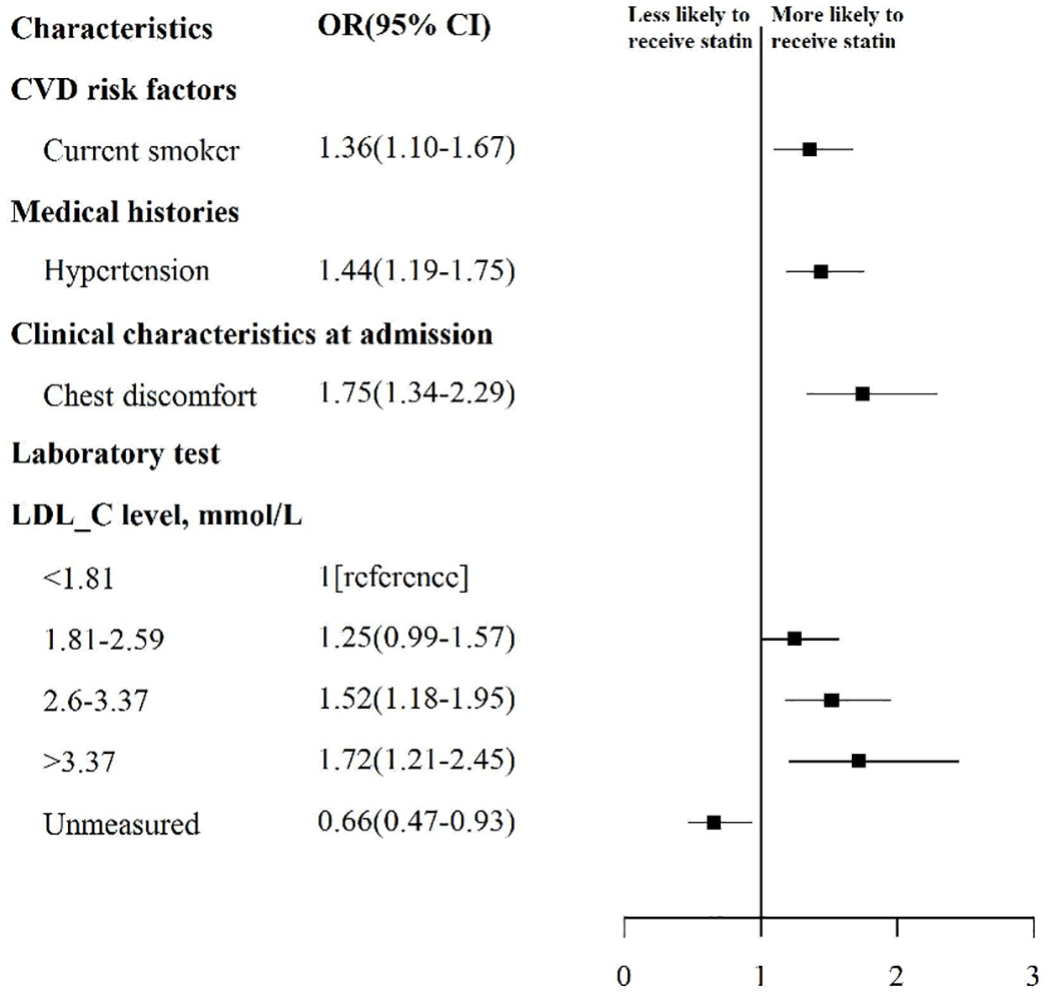
Factors Independently Associated with Statin Use in 2011. Variables with significant association with any usage of statin are shown along the vertical axis. The strength of effect is shown along the horizontal axis with the vertical dotted line demarking an odds ratio of 1 (that is, no association); estimates to the right (that is, > 1) are associated with greater likelihood of early statin use, while those to the left (that is, < 1) indicate association with reduced likelihood of early statin use. Each square and line represents the point estimate of the effect of that variable in the model, while the line shows the 95% confidence interval. CVD indicates cardiovascular disease.

In the multivariable model, several patient and hospital factors were associated with any statin therapy in 2011 (Fig 5). Patients whose LDL-C was not measured during the hospitalization were significantly less likely to receive statin therapy (OR = 0.66; 95% CI 0.47–0.93, P<0.001), patients with LDL-C 2.6–3.37 mmol/L (OR = 1.52; 95%CI 1.18–1.95, P<0.001) or LDL-C >3.37 mmol/L (OR = 1.72; 95% CI 1.21–2.45, P<0.001) were more likely to receive statin therapy than those with LDL-C<1.81mmol/L. Smoking and hypertension were also associated with more likely to receive statin therapy.

### Factors associated with use of intensive statin therapy in 2011

**Fig 6:**
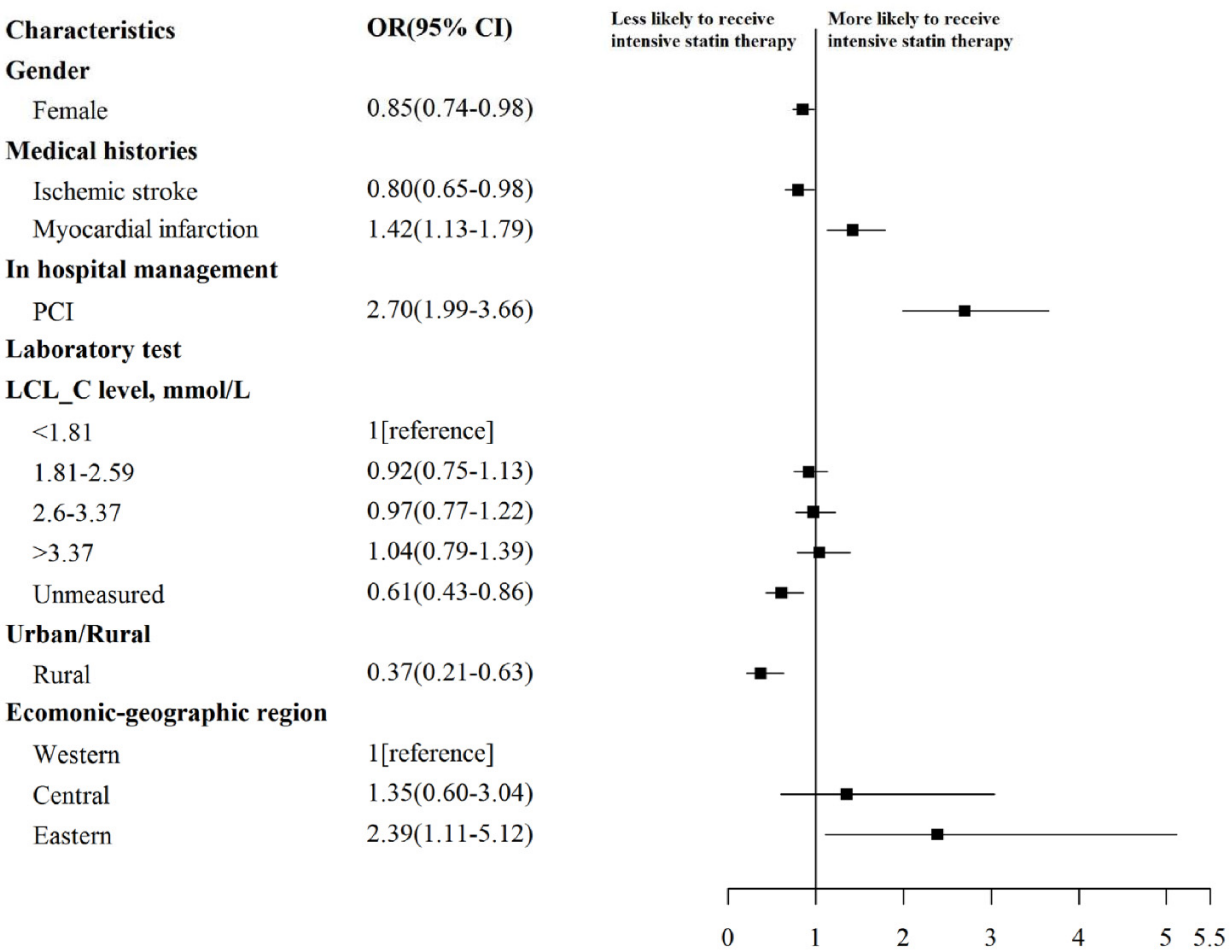
Factors Independently Associated with Intensive Statin Therapy in 2011. Variables with significant association with usage of intensive statin therapy are shown along the vertical axis. The strength of effect is shown along the horizontal axis with the vertical dotted line demarking an odds ratio of 1 (that is, no association); estimates to the right (that is, >1) are associated with greater likelihood of early statin use, while those to the left (that is, <1) indicate association with reduced likelihood of early statin use. Each square and line represents the point estimate of the effect of that variable in the model, while the line shows the 95% confidence interval.

In the multivariable model including patients receiving any statin therapy(Fig 6), intensive therapy was used less often in women (OR = 0.85; 95%CI 0.74–0.98, P<0.001), patients with prior ischemic stroke(OR = 0.80; 95%CI 0.65–0.98, P<0.001) and patients without LDL-C measured (OR = 0.61; 95%CI 0.43–0.86, P<0.001). In contrast, patients receiving PCI were more likely to receive intensity statin therapy (OR = 2.70; 95%CI 1.99–3.66, P<0.001).

After accounting for differences in patient factors, those hospitalized in rural hospitals were also less likely to receive intensive statin therapy compared with those in urban hospitals(OR = 0.37;95%CI 0.21–0.63, P<0.001). In addition, patients in Eastern region (the most developed region in China) were more likely to receive intensive statin therapy (OR = 2.39; 95% CI 1.11–5.12, P<0.001) compared with those in Western region.

## Discussion

In this first national quality assessment of statin use among hospitalized patients with AMI in China, we found a substantial increase in the use of these drugs among patients with AMI over the past decade. However, there is still a substantial opportunity for further improvement because 10% of eligible patients were not treated with these potentially life-saving drugs in 2011. Additionally, among patients receiving statin therapy, almost half did not receive intensive therapy as defined by Chinese practice guidelines. While there were gains in both rural and urban regions, as well as all economic-geographic regions, notable variation persisted in 2011. Notably, patients receiving care in rural hospitals were significantly less likely to receive a statin of any intensity.

The increase in statin use from 2001 and 2011 in China represents a substantial improvement in the use of evidence-based care for patients with AMI. Several factors may have driven this improvement. For example, accumulating evidence of benefit of statins led codification of statins use in guidelines [8,11, 20–21],and an increased awareness of the fact that statins are simple and generally safe. In addition, the increasing coverage provided by medical insurance programs and the decrease in out-of-pocket payment for statins may also contribute to the improvements observed in our study [22, 23].

Despite high rates of statin use, intensive statin regimens, which are recommended by many guidelines, were only used in just over half of patients with AMI in China in 2011. Similar gaps have also been observed in the US [24], France [25], and Canada [26], although the higher threshold for LDL-C lowering of 50% was used to defined intensive therapy in these countries. Potential barriers to the greater use of intensive statin regimens could include physicians’ concerns about dose-dependent adverse effects of statin [27, 28], an especially pertinent consideration given the increased risk of adverse events among Chinese patients [14]. Moreover, previous studies have shown that Asian patients need lower statin dosage to achieve the same LDL-lowering efficacy [29], and the highest approved dosages of some statins are lower in China than Western countries. Although these factors may discourage the use of higher dose statin, we defined intensive therapy based upon the Chinese guidelines, which are more conservative than US guidelines. Finally, the challenges in the structure of healthcare systems might create barriers that impede best practice,. The suboptimal use of intensive statin therapy highlights an important opportunity to improve care and reduce disparities.

Additionally, certain patient groups were particularly likely to be untreated or under-treated. Women were less likely to be treated with intensive statin therapy, a finding consistent with a recent analysis in the United States [30]. Since some studies have shown that women may experience more side effects from statin therapy compared with men [31, 32], the sex differences observed in our study may reflect physician’s concerns about the risks associated with statin therapy. Patients with a history of ischemic stroke were less likely to receive intensive statin therapy, which was consistent with a previous study report in China [33]. This may reflect concerns about the possible association between statin therapy and hemorrhagic stroke, the incidence of which is much higher in China than in the West [34]. Additionally, those without LDL-C measurement were much less likely to receive any statin therapy as well intensive statin therapy, a finding consistent with previous studies [35,36].Taken together with our complementary finding that those with higher LDL-C were more likely to receive statin therapy, our study supports the hypothesis that physicians’ decisions about statin use are still guided by measurements of LDL-C, a practice that is not now endorsed by guidelines [37]. Educational efforts are needed to emphasize the benefits of statin therapy for all patients with AMI who tolerate this treatment and to construct performance measures to support broader and more consistent adoption of current guidelines for statin therapy.

While hospitals across all economic-geographical regions improved, disparities between them persisted: patients treated in rural centers were less likely to receive intensive statin therapy in 2011. A number of factors may explain this observation. Differences in per capita income, which are marked between urban and rural, are likely to influence the utilization of medical resources. Despite health care reforms, medical insurance still does not cover the full cost of statin therapy [23], leaving patients with lower income at particular risk for under-use. Another explanation for the differences in statin utilization according to hospital location is that many urban hospitals are staffed with cardiovascular specialists [16],and the availability of practitioners with greater condition-specific expertise may explain higher rates of statin use. In contrast, most rural hospitals, which together are responsible for the care of more than half of China’s population, have limited clinical capacity for advanced cardiac care and limited funding [16]. Initiatives to improve statin utilization that emphasize rural hospitals could have an important impact on the disparities noted in this national quality assessment.

This is the first nationally representative study evaluating trends and disparities for statin utilization among patients with AMI hospitalized in China. The findings of our study have important implications for efforts to understand past performance and to improve the quality of AMI care in China. These findings will serve as the foundation for future national quality improvement efforts to overcome the barriers for the appropriate use of intensive statin therapy.

Several limitations of our study should be noted. First, as with all retrospective studies, it was limited by the quality of documentation in the medical records. To ensure our approach to data collection was accurate, we independently re-abstracted 5% medical records noting accuracy rates exceeding 98% and giving us great confidence in the accuracy of the abstraction process. Second, it is possible that our approach underestimated the number of patients with contraindications to statin therapy because there may have been poor documentation of contraindication in the medical chart, which could indicate another opportunity to improve the transparency and quality of care. Third, we did not collect the information about statin therapy before admission, which might influence decisions around statin use and dosing. Finally, we did not collect other factors, such as physicians’ attitude about intensive statin therapy or patients’ refusal of treatments, which have the potential to influence rates of statin utilization.

## Conclusion

This national quality assessment found that the use of statin among patients with AMI in China increased sharply over the past decade; however, several opportunities to improve care persist, particularly with regards to the use of intensive statin therapy. Our findings highlight an opportunity for better translation of evidence into clinical practice with a focus on more consistent care throughout China.

## Supporting Information

S1 Appendix China PEACE-Retrospective AMI Study Site Investigators by Hospital. (DOCX)

S2 Appendix China PEACE Study Consultants. (DOCX)

S3 Appendix Procedures to Identify Factors Independently Associated with Statin Use in 2011. (DOCX)

S4 Appendix Procedures to Identify Factors Independently Associated with Intensive Statin Use in 2011. (DOCX)

S1 Fig Temporal Trends in Statin Therapy according to LDL-C Level. (TIF)

S2 Fig Proportion of Each Type of Statin Agents in 2001, 2006, 2011. (TIF)

S1 Table Definition of statin equivalency (intensive statin therapy highlighted). (DOCX)
