# Supplementary material for: National Assessment of Statin Therapy in Patients Hospitalized with Acute Myocardial Infarction: Insight from China PEACE-Retrospective AMI Study, 2001, 2006, 2011
Source: PLoS One. 2016 Apr 8;11(4):e0150806. doi: 10.1371/journal.pone.0150806 (PMC4825974; doi:10.1371/journal.pone.0150806)
Supplement: S2 Appendix — (DOCX) [file pone.0150806.s002.docx]

**China PEACE Study Consultants**

Study Consultants: Paul S. Chan, MD, MSc, Jersey Chen, MD, MPH, David J. Cohen, MD, MSc, Nihar R. Desai, MD, MPH, Kumar Dharmarajan MD, MBA, Mikhail N. Kosiborod, MD, Jing Li, MD, PhD, Xi Li, MD, PhD, Zhenqiu Lin, PhD, Frederick A. Masoudi, MD, MSPH, Jennifer Mattera, DrPH, MPH, Brahmajee K. Nallamothu, MD, MPH, Khurram Nasir, MD, MPH, Sharon-Lise T. Normand, PhD, Joseph S. Ross, MD MHS, John A. Spertus, MD, MPH, Henry H. Ting, MD, Xiao Xu, PhD

St. Luke’s Mid America Heart Institute/University of Missouri Kansas City (PSC, DJC, MNK, JAS), Kansas City, Missouri, United States; Kaiser Permanente (JC), Mid-Atlantic Permanente Research Institute, Rockville, Maryland, United States; Center for Outcomes Research and Evaluation (NRD, KD, ZL, JM, JSR, XX), Yale-New Haven Hospital, New Haven, Connecticut, United States; Division of Cardiology (KD), Department of Internal Medicine, Columbia University Medical Center, New York, New York, United States; State Key Laboratory of Cardiovascular Disease (JL, XL), China Oxford Centre for International Health Research, Fuwai Hospital, National Center for Cardiovascular Diseases, Chinese Academy of Medical Sciences and Peking Union Medical College, Beijing, People's Republic of China; Division of Cardiology (FAM), University of Colorado Anschutz Medical Campus, Aurora, Colorado, United States; Veterans Affairs Health Services Research and Development Center of Excellence (BKN), Veterans Affairs Ann Arbor Healthcare System, Ann Arbor, Michigan, United States; Department of Internal Medicine (BKN) and Center for Healthcare Outcomes and Policy (BKN), University of Michigan, Ann Arbor, Michigan, United States; Research Director, Center for Prevention and Wellness (KN), Baptist Health South Florida, Miami, Florida, United States; Department of Biostatistics (S-LTN), Harvard School of Public Health, Boston, Massachusetts, United States; Department of Health Care Policy (S-LTN), Harvard Medical School, Boston, Massachusetts, United States; Section of General Internal Medicine and the Robert Wood Johnson Clinical Scholars Program (JSR), Department of Internal Medicine, Yale University School of Medicine, Connecticut, United States; Division of Cardiovascular Diseases (HHT) and Knowledge and Evaluation Research Unit (HHT), Mayo Clinic College of Medicine, Rochester, Minnesota. United States; Department of Obstetrics, Gynecology, and Reproductive Sciences (XX), Yale School of Medicine, New Haven, Connecticut, United States
