# Supplementary material for: National Assessment of Statin Therapy in Patients Hospitalized with Acute Myocardial Infarction: Insight from China PEACE-Retrospective AMI Study, 2001, 2006, 2011
Source: PLoS One. 2016 Apr 8;11(4):e0150806. doi: 10.1371/journal.pone.0150806 (PMC4825974; doi:10.1371/journal.pone.0150806)
Supplement: S3 Appendix — (DOCX) [file pone.0150806.s003.docx]

**Procedures to Identify Factors Independently Associated with Statin Use in 2011**

Step 1

| Variables | Odds ratio | 95% Confidence interval | P value |
| --- | --- | --- | --- |
| Demographic |  |  |  |
| Age, years |  |  |  |
| Age<55 | 1 | 1 |  |
| Age 55-64 | 0.89 | 0.73-1.07 | 0.204 |
| Age 65-74 | 0.98 | 0.8-1.2 | 0.860 |
| Age≥75 | 0.96 | 0.76-1.21 | 0.713 |
| Gender |  |  |  |
| Male | 1 | 1 |  |
| Female | 0.96 | 0.8-1.14 | 0.96 |
| Cardiovascular risk factors |  |  |  |
| Hypertension | 1.35 | 1.11-1.65 | 0.003 |
| Diabetes | 1.15 | 0.93-1.41 | 0.197 |
| Dyslipidemia | 1.2 | 0.98-1.46 | 0.081 |
| Current smoker | 1.22 | 0.98-1.52 | 0.073 |
| Medical histories |  |  |  |
| Myocardial infarction | 0.99 | 0.71-1.38 | 0.954 |
| Ischemic stroke | 0.86 | 0.65-1.15 | 0.310 |
| Hemorrhagic stroke | 0.81 | 0.46-1.42 | 0.465 |
| Clinical characteristics at admission |  |  |  |
| Chest discomfort | 1.63 | 1.28-2.07 | <.0001 |
| Cardiac arrest | 1.23 | 0.61-2.45 | 0.563 |
| Cardiac shock | 0.91 | 0.64-1.28 | 0.586 |
| AMI type |  |  |  |
| NSTEMI | 1 | 1 |  |
| STEMI | 1.14 | 0.93-1.4 | 0.196 |
| In hospital management |  |  |  |
| PCI | 1.54 | 0.56-4.23 | 0.407 |
| LDL-C level, mmol/L |  |  |  |
| LDL-C<1.81 | 1 | 1 |  |
| LDL-C:1.81-2.59 | 1.23 | 0.97-1.55 | 0.091 |
| LDL-C:2.6-3.37 | 1.42 | 1.12-1.79 | 0.004 |
| LDL-C> 3.37 | 1.5 | 1.08-2.1 | 0.017 |
| LDL-C Unrecorded | 0.75 | 0.54-1.05 | 0.092 |
| Region |  |  |  |
| Western | 1 | 1 |  |
| Eastern | 1.23 | 0.37-4.07 | 0.736 |
| Central | 0.96 | 0.32-2.87 | 0.946 |
| Urban/Rural |  |  |  |
| Urban | 1 | 1 |  |
| Rural | 0.60 | 0.36-1.01 | 0.055 |

Step 2

| Variables | Odds ratio | 95% Confidence interval | P value |
| --- | --- | --- | --- |
| Demographic |  |  |  |
| Age, years |  |  |  |
| Age<55 | 1 | 1 |  |
| Age55-64 | 0.89 | 0.73-1.07 | 0.204 |
| Age:65-74 | 0.98 | 0.80-1.20 | 0.861 |
| Age≥75 | 0.96 | 0.76-1.20 | 0.712 |
| Gender |  |  |  |
| Male | 1 | 1 |  |
| Female | 0.96 | 0.80-1.14 | 0.616 |
| Cardiovascular risk factors |  |  |  |
| Hypertension | 1.35 | 1.11-1.65 | 0.002 |
| Diabetes | 1.15 | 0.93-1.41 | 0.198 |
| Dyslipidemia | 1.20 | 0.98-1.46 | 0.082 |
| Current smoker | 1.22 | 0.98-1.52 | 0.073 |
| Medical histories |  |  |  |
| Ischemic stroke | 0.86 | 0.65-1.15 | 0.309 |
| Hemorrhagic stroke | 0.81 | 0.46-1.42 | 0.465 |
| Clinical characteristics at admission |  |  |  |
| Chest discomfort | 1.63 | 1.28-2.07 | <.0001 |
| Cardiac arrest | 1.23 | 0.61-2.45 | 0.563 |
| Cardiac shock | 0.91 | 0.64-1.28 | 0.585 |
| AMI type |  |  |  |
| NSTEMI | 1 | 1 |  |
| STEMI | 1.14 | 0.93-1.40 | 0.199 |
| In hospital management |  |  |  |
| PCI | 1.54 | 0.56-4.23 | 0.407 |
| LDL-C level, mmol/L |  |  |  |
| LDL-C<1.81 | 1 | 1.00-1.00 |  |
| LDL-C:1.81-2.59 | 1.23 | 0.97-1.55 | 0.091 |
| LDL-C:2.6-3.37 | 1.42 | 1.12-1.80 | 0.004 |
| LDL-C> 3.37 | 1.50 | 1.08-2.10 | 0.017 |
| LDL-C Unrecorded | 0.75 | 0.54-1.05 | 0.091 |
| Economic-geographic region |  |  |  |
| Western | 1 | 1 |  |
| Eastern | 1.23 | 0.37-4.06 | 0.735 |
| Central | 0.96 | 0.32-2.87 | 0.946 |
| Urban/Rural |  |  |  |
| Urban | 1 | 1 |  |
| Rural | 0.60 | 0.36-1.01 | 0.055 |

Step 3

| Variables | | Odds ratio | 95% Confidence interval | | P value |
| --- | --- | --- | --- | --- | --- |
| Demographic | |  |  | |  |
| Age, years | |  |  | |  |
| Age<55 | | 1 | 1 | |  |
| Age 55-64 | | 0.89 | 0.74-1.07 | | 0.213 |
| Age 65-74 | | 0.99 | 0.81-1.21 | | 0.914 |
| Age≥75 | | 0.98 | 0.79-1.21 | | 0.849 |
| Gender | |  |  | |  |
| Male | | 1 | 1 | |  |
| Female | | 0.96 | 0.80-1.15 | | 0.681 |
| Cardiovascular risk factors | |  |  | |  |
| Hypertension | | 1.37 | 1.14-1.64 | | 0.001 |
| Diabetes | | 1.17 | 0.95-1.42 | | 0.134 |
| Dyslipidemia | | 1.21 | 0.98-1.49 | | 0.075 |
| Current smoker | | 1.23 | 0.98-1.56 | | 0.079 |
| Medical histories | |  |  | |  |
| Ischemic stroke | | 0.88 | 0.65-1.19 | | 0.406 |
| Hemorrhagic stroke | | 0.82 | 0.46-1.46 | | 0.503 |
| Clinical characteristics at admission |  | | |  |  |
| Chest discomfort | | 1.62 | 1.28-2.06 | | <.0001 |
| Cardiac arrest | | 1.24 | 0.59-2.60 | | 0.574 |
| Cardiac shock | | 0.91 | 0.64-1.28 | | 0.583 |
| AMI type | |  |  | |  |
| NSTEMI | | 1 | 1 | |  |
| STEMI | | 1.14 | 0.93-1.40 | | 0.219 |
| In hospital management | |  |  | |  |
| PCI | | 1.57 | 0.63-3.97 | | 0.335 |
| LDL-C level, mmol/L | |  |  | |  |
| LDL-C<1.81 | | 1 | 1 | |  |
| LDL-C:1.81-2.59 | | 1.24 | 0.98-1.56 | | 0.072 |
| LDL-C:2.6-3.37 | | 1.44 | 1.13-1.84 | | 0.004 |
| LDL-C> 3.37 | | 1.55 | 1.11-2.15 | | 0.009 |
| LDL-C Unrecorded | | 0.76 | 0.54-1.08 | | 0.131 |
| Urban/Rural | |  |  | |  |
| Urban | | 1 | 1 | |  |
| Rural | | 0.59 | 0.33-1.04 | | 0.066 |

Step 4

| Variables | Odds ratio | 95% Confidence interval | P value |
| --- | --- | --- | --- |
| Demographic |  |  |  |
| Age, years |  |  |  |
| Age<55 | 1 | 1 |  |
| Age 55-64 | 0.89 | 0.74-1.07 | 0.229 |
| Age 65-74 | 0.99 | 0.81-1.22 | 0.956 |
| Age≥75 | 0.99 | 0.80-1.22 | 0.898 |
| Gender |  |  |  |
| Male | 1 | 1 |  |
| Female | 0.96 | 0.80-1.15 | 0.664 |
| Cardiovascular risk factors |  |  |  |
| Hypertension | 1.37 | 1.15-1.64 | 0.001 |
| Diabetes | 1.17 | 0.96-1.42 | 0.116 |
| Dyslipidemia | 1.21 | 0.98-1.49 | 0.074 |
| Current smoker | 1.23 | 0.98-1.55 | 0.079 |
| Medical histories |  |  |  |
| Ischemic stroke | 0.88 | 0.65-1.20 | 0.413 |
| Hemorrhagic stroke | 0.82 | 0.46-1.45 | 0.496 |
| Clinical characteristics at admission | |  |  |
| Chest discomfort | 1.63 | 1.28-2.07 | <.0001 |
| Cardiac arrest | 1.24 | 0.59-2.60 | 0.574 |
| Cardiac shock | 0.91 | 0.64-1.28 | 0.585 |
| AMI type |  |  |  |
| NSTEMI | 1 | 1 |  |
| STEMI | 1.13 | 0.92-1.39 | 0.196 |
| In hospital management |  |  |  |
| PCI | 1.57 | 0.62-3.98 | 0.407 |
| LDL-C level, mmol/L |  |  |  |
| LDL-C<1.81 | 1 | 1 |  |
| LDL-C:1.81-2.59 | 1.24 | 0.98-1.56 | 0.074 |
| LDL-C:2.6-3.37 | 1.44 | 1.13-1.83 | 0.004 |
| LDL-C> 3.37 | 1.55 | 1.11-2.15 | 0.010 |
| LDL-C Unrecorded | 0.76 | 0.54-1.08 | 0.130 |
| Urban/rural  Urban | 1 | 1 |  |
| Rural | 0.59 | 0.33-1.03 | 0.065 |

Step 5

| Variables | Odds ratio | 95% Confidence interval | P value |
| --- | --- | --- | --- |
| Demographic |  |  |  |
| Age, years |  |  |  |
| Age<55 | 1 | 1 |  |
| Age: 55-64 | 0.89 | 0.74-1.07 | 0.218 |
| Age:65-74 | 0.99 | 0.80-1.21 | 0.906 |
| Age≥75 | 0.98 | 0.79-1.20 | 0.830 |
| Cardiovascular risk factors |  |  |  |
| Hypertension | 1.37 | 1.15-1.63 | <0.001 |
| Diabetes | 1.16 | 0.96-1.41 | 0.123 |
| Dyslipidemia | 1.20 | 0.98-1.48 | 0.080 |
| Current smoker | 1.24 | 1.00-1.55 | 0.053 |
| Medical histories |  |  |  |
| Ischemic stroke | 0.88 | 0.65-1.19 | 0.417 |
| Hemorrhagic stroke | 0.82 | 0.47-1.45 | 0.499 |
| Clinical characteristics at admission | |  |  |
| Chest discomfort | 1.63 | 1.28-2.07 | <0.001 |
| Cardiac arrest | 1.24 | 0.59-2.61 | 0.574 |
| Cardiac shock | 0.91 | 0.64-1.28 | 0.578 |
| AMI type |  |  |  |
| NSTEMI | 1 | 1 |  |
| STEMI | 1.14 | 0.92-1.39 | 0.223 |
| In hospital management |  |  |  |
| PCI | 1.57 | 0.62-3.99 | 0.339 |
| LDL-C level, mmol/L |  |  |  |
| LDL-C<1.81 | 1 | 1 |  |
| LDL-C:1.81-2.59 | 1.24 | 0.98-1.56 | 0.076 |
| LDL-C:2.6-3.37 | 1.44 | 1.13-1.83 | 0.004 |
| LDL-C> 3.37 | 1.55 | 1.11-2.15 | 0.010 |
| LDL-C Unrecorded | 0.76 | 0.54-1.08 | 0.127 |
| Urban/Rural |  |  |  |
| Urban | 1 | 1 |  |
| Rural | 0.59 | 0.33-1.03 | 0.065 |

Step 6

| Variables | Odds ratio | 95% Confidence interval | P value |
| --- | --- | --- | --- |
| Demographic |  |  |  |
| Age, years |  |  |  |
| Age<55 | 1 | 1 |  |
| Age: 55-64 | 0.89 | 0.74-1.07 | 0.214 |
| Age:65-74 | 0.99 | 0.80-1.21 | 0.886 |
| Age≥75 | 0.97 | 0.79-1.20 | 0.806 |
| Cardiovascular risk factors |  |  |  |
| Hypertension | 1.37 | 1.15-1.63 | <0.001 |
| Diabetes | 1.16 | 0.96-1.41 | 0.125 |
| Dyslipidemia | 1.20 | 0.98-1.48 | 0.080 |
| Current smoker | 1.24 | 1.00-1.55 | 0.054 |
| Medical histories |  |  |  |
| Ischemic stroke | 0.88 | 0.65-1.19 | 0.416 |
| Hemorrhagic stroke | 0.82 | 0.47-1.45 | 0.501 |
| Clinical characteristics at admission | |  |  |
| Chest discomfort | 1.63 | 1.28-2.07 | <.0001 |
| Cardiac arrest | 1.21 | 0.57-2.55 | 0.616 |
| AMI type |  |  |  |
| NSTEMI | 1 | 1 |  |
| STEMI | 1.13 | 0.92-1.39 | 0.233 |
| In hospital management |  |  |  |
| PCI | 1.58 | 0.62-3.99 | 0.338 |
| LDL-C level, mmol/L |  |  |  |
| LDL-C<1.81 | 1 | 1 |  |
| LDL-C:1.81-2.59 | 1.24 | 0.98-1.56 | 0.073 |
| LDL-C:2.6-3.37 | 1.44 | 1.13-1.83 | 0.004 |
| LDL-C> 3.37 | 1.54 | 1.11-2.14 | 0.010 |
| LDL-C Unrecorded | 0.76 | 0.54-1.08 | 0.127 |
| Urban/Rural |  |  |  |
| Urban | 1 | 1 |  |
| Rural | 0.59 | 0.33-1.03 | 0.065 |

Step 7

| Variables | Odds ratio | 95% Confidence interval | P value |
| --- | --- | --- | --- |
| Demographic |  |  |  |
| Age, years |  |  |  |
| Age<55 | 1 | 1 |  |
| Age: 55-64 | 0.89 | 0.74-1.07 | 0.211 |
| Age:65-74 | 0.98 | 0.80-1.21 | 0.874 |
| Age≥75 | 0.97 | 0.79-1.20 | 0.797 |
| Cardiovascular risk factors |  |  |  |
| Hypertension | 1.37 | 1.15-1.63 | <0.0001 |
| Diabetes | 1.16 | 0.96-1.41 | 0.127 |
| Dyslipidemia | 1.20 | 0.98-1.48 | 0.079 |
| Current smoker | 1.24 | 1.00-1.55 | 0.054 |
| Medical histories |  |  |  |
| Ischemic stroke | 0.88 | 0.65-1.19 | 0.409 |
| Hemorrhagic stroke | 0.82 | 0.47-1.45 | 0.496 |
| Clinical characteristics at admission | |  |  |
| Chest discomfort | 1.63 | 1.28-2.07 | <0.0001 |
| AMI type |  |  |  |
| NSTEMI | 1 | 1 |  |
| STEMI | 1.13 | 0.92-1.39 | 0.229 |
| In hospital management |  |  |  |
| PCI | 1.58 | 0.62-3.99 | 0.336 |
| LDL-C level, mmol/L |  |  |  |
| LDL-C<1.81 | 1 | 1 |  |
| LDL-C:1.81-2.59 | 1.23 | 0.98-1.56 | 0.077 |
| LDL-C:2.6-3.37 | 1.43 | 1.12-1.83 | 0.004 |
| LDL-C> 3.37 | 1.54 | 1.11-2.14 | 0.010 |
| LDL-C Unrecorded | 0.76 | 0.54-1.08 | 0.126 |
| Urban/Rural |  |  |  |
| Urban | 1 | 1 |  |
| Rural | 0.59 | 0.33-1.03 | 0.063 |

Step 8

| Variables | Odds ratio | 95% Confidence interval | | P value | |
| --- | --- | --- | --- | --- | --- |
| Demographic |  |  | | |  |
| Age, years |  |  | | |  |
| Age<55 | 1 | 1 | | |  |
| Age: 55-64 | 0.89 | 0.74-1.07 | | | 0.203 |
| Age:65-74 | 0.98 | 0.80-1.21 | | | 0.871 |
| Age≥75 | 0.97 | 0.79-1.20 | | | 0.807 |
| Cardiovascular risk factors |  |  | | |  |
| Hypertension | 1.37 | 1.15-1.62 | | | <0.0001 |
| Diabetes | 1.16 | 0.96-1.41 | | | 0.125 |
| Dyslipidemia | 1.20 | 0.98-1.48 | | | 0.077 |
| Current smoker | 1.25 | 1.00-1.55 | | | 0.051 |
| Medical histories |  |  | | |  |
| Ischemic stroke | 0.88 | 0.65-1.19 | | | 0.394 |
| Clinical characteristics at admission | |  | | |  |
| Chest discomfort | 1.63 | 1.28-2.07 | | | <0.0001 |
| AMI type |  |  | | |  |
| NSTEMI | 1 | 1 | | |  |
| STEMI | 1.13 | 0.92-1.39 | | | 0.234 |
| In hospital management |  |  | | |  |
| PCI | 1.58 | 0.62-3.99 | | | 0.336 |
| LDL-C level, mmol/L |  |  | | |  |
| LDL-C<1.81 | 1 | 1 | | |  |
| LDL-C:1.81-2.59 | 1.23 | | 0.98-1.56 | | 0.077 |
| LDL-C:2.6-3.37 | 1.43 | | 1.12-1.83 | | 0.004 |
| LDL-C> 3.37 | 1.54 | | 1.11-2.14 | | 0.011 |
| LDL-C Unrecorded | 0.76 | | 0.53-1.08 | | 0.126 |
| Urban/Rural |  | |  | |  |
| Urban | 1 | | 1 | |  |
| Rural | 0.59 | | 0.34-1.03 | | 0.063 |

Step 9

| Variables | Odd ratio | 95% Confidence interval | P value |
| --- | --- | --- | --- |
| Demographic |  |  |  |
| Age, years |  |  |  |
| Age<55 | 1 | 1 |  |
| Age: 55-64 | 0.88 | 0.73-1.06 | 0.184 |
| Age:65-74 | 0.97 | 0.80-1.20 | 0.798 |
| Age≥75 | 0.96 | 0.78-1.19 | 0.724 |
| Cardiovascular risk factors |  |  |  |
| Hypertension | 1.35 | 1.14-1.61 | 0.001 |
| Diabetes | 1.15 | 0.95-1.40 | 0.149 |
| Dyslipidemia | 1.20 | 0.98-1.48 | 0.077 |
| Current smoker | 1.25 | 1.00-1.55 | 0.052 |
| Clinical characteristics at admission | |  |  |
| Chest discomfort | 1.65 | 1.30-2.09 | <0.0001 |
| AMI type |  |  |  |
| NSTEMI | 1 | 1 |  |
| STEMI | 1.13 | 0.92-1.39 | 0.238 |
| In hospital management |  |  |  |
| PCI | 1.58 | 0.62-4.01 | 0.334 |
| LDL-C level, mmol/L |  |  |  |
| LDL-C<1.81 | 1 | 1 |  |
| LDL-C:1.81-2.59 | 1.23 | 0.98-1.55 | 0.078 |
| LDL-C:2.6-3.37 | 1.43 | 1.12-1.82 | 0.004 |
| LDL-C> 3.37 | 1.53 | 1.11-2.13 | 0.011 |
| LDL-C Unrecorded | 0.76 | 0.53-1.08 | 0.122 |
| Urban/Rural |  |  |  |
| Urban | 1 | 1 |  |
| Rural | 0.59 | 0.34-1.03 | 0.063 |

Step 10

| Variables | Odds ratio | 95% Confidence interval | P value |
| --- | --- | --- | --- |
| Demographic |  |  |  |
| Age, years |  |  |  |
| Age<55 | 1 | 1 |  |
| Age: 55-64 | 0.88 | 0.73-1.06 | 0.181 |
| Age:65-74 | 0.95 | 0.76-1.17 | 0.617 |
| Age≥75 | 0.91 | 0.73-1.14 | 0.403 |
| Cardiovascular risk factors |  |  |  |
| Hypertension | 1.35 | 1.14-1.61 | 0.001 |
| Diabetes | 1.14 | 0.94-1.39 | 0.183 |
| Dyslipidemia | 1.21 | 0.99-1.49 | 0.069 |
| Current smoker | 1.27 | 1.03-1.56 | 0.024 |
| Clinical characteristics at admission | |  |  |
| Chest discomfort | 1.72 | 1.35-2.21 | <0.0001 |
| AMI type |  |  |  |
| NSTEMI | 1 | 1 |  |
| STEMI | 1.16 | 0.94-1.44 | 0.162 |
| LDL-C level, mmol/L |  |  |  |
| LDL-C<1.81 | 1 | 1 |  |
| LDL-C:1.81-2.59 | 1.26 | 0.99-1.60 | 0.063 |
| LDL-C:2.6-3.37 | 1.46 | 1.15-1.87 | 0.002 |
| LDL-C> 3.37 | 1.56 | 1.15-2.20 | 0.010 |
| LDL-C Unrecorded | 0.76 | 0.53-1.08 | 0.122 |
| Urban/Rural |  |  |  |
| Urban | 1 | 1 |  |
| Rural | 0.53 | 0.27-1.02 | 0.056 |

Step 11

| Variables | Odds ratio | 95% Confidence interval | P value |
| --- | --- | --- | --- |
| Demographic |  |  |  |
| Age, years |  |  |  |
| Age<55 | 1 | 1 |  |
| Age: 55-64 | 0.88 | 0.73-1.07 | 0.196 |
| Age:65-74 | 0.95 | 0.76-1.18 | 0.645 |
| Age≥75 | 0.91 | 0.73-1.14 | 0.406 |
| Cardiovascular risk factors |  |  |  |
| Hypertension | 1.37 | 1.15-1.63 | <0.0001 |
| Dyslipidemia | 1.23 | 0.99-1.51 | 0.057 |
| Current smoker | 1.26 | 1.03-1.54 | 0.027 |
| Clinical characteristics at admission | |  |  |
| Chest discomfort | 1.71 | 1.35-2.19 | <0.0001 |
| AMI type |  |  |  |
| NSTEMI | 1 | 1 |  |
| STEMI | 1.16 | 0.93-1.44 | 0.182 |
| LDL-C level, mmol/L |  |  |  |
| LDL-C<1.81 | 1 | 1 |  |
| LDL-C:1.81-2.59 | 1.26 | 0.99-1.60 | 0.062 |
| LDL-C:2.6-3.37 | 1.47 | 1.15-1.87 | 0.002 |
| LDL-C> 3.37 | 1.56 | 1.11-2.19 | 0.011 |
| LDL-C Unrecorded | 0.76 | 0.53-1.08 | 0.130 |
| Urban/Rural |  |  |  |
| Urban | 1 | 1 |  |
| Rural | 0.52 | 0.27-1.01 | 0.052 |

Step 12

| Variables | Odds ratio | 95% Confidence interval | P value |
| --- | --- | --- | --- |
| Cardiovascular risk factors |  |  |  |
| Hypertension | 1.37 | 1.14-1.63 | 0.001 |
| Dyslipidemia | 1.23 | 1.00-1.52 | 0.052 |
| Current smoker | 1.27 | 1.05-1.54 | 0.014 |
| Clinical characteristics at admission | |  |  |
| Chest discomfort | 1.72 | 1.31-2.24 | <0.0001 |
| AMI type |  |  |  |
| NSTEMI | 1 | 1 |  |
| STEMI | 1.16 | 0.94-1.44 | 0.171 |
| LDL-C level, mmol/L |  |  |  |
| LDL-C<1.81 | 1 | 1 |  |
| LDL-C:1.81-2.59 | 1.26 | 0.99-1.61 | 0.061 |
| LDL-C:2.6-3.37 | 1.47 | 1.15-1.87 | 0.002 |
| LDL-C> 3.37 | 1.57 | 1.11-2.20 | 0.010 |
| LDL-C Unrecorded | 0.76 | 0.53-1.08 | 0.128 |
| Urban/Rural |  |  |  |
| Urban | 1 | 1 |  |
| Rural | 0.52 | 0.27-1.00 | 0.051 |

Step 13

| Variables | Odds ratio | 95% Confidence interval | P value |
| --- | --- | --- | --- |
| Cardiovascular risk factors |  |  |  |
| Hypertension | 1.35 | 1.13-1.61 | 0.001 |
| Dyslipidemia | 1.23 | 1.00-1.52 | 0.052 |
| Current smoker | 1.28 | 1.06-1.55 | 0.011 |
| Clinical characteristics at admission | |  |  |
| Chest discomfort | 1.74 | 1.33-2.27 | <0.0001 |
| LDL-C level, mmol/L |  |  |  |
| LDL-C<1.81 | 1 | 1 |  |
| LDL-C:1.81-2.59 | 1.26 | 0.99-1.60 | 0.063 |
| LDL-C:2.6-3.37 | 1.46 | 1.15-1.87 | 0.002 |
| LDL-C> 3.37 | 1.56 | 1.11-2.18 | 0.010 |
| LDL-C Unrecorded | 0.76 | 0.53-1.08 | 0.122 |
| Urban/Rural |  |  |  |
| Urban | 1 | 1 |  |
| Rural | 0.52 | 0.27-1.01 | 0.052 |

Step 14

| Variables | Odds ratio | 95% Confidence interval | P value |
| --- | --- | --- | --- |
| Cardiovascular risk factors |  |  |  |
| Hypertension | 1.37 | 1.15-1.63 | <0.0001 |
| Current smoker | 1.28 | 1.06-1.55 | 0.012 |
| Clinical characteristics at admission | |  |  |
| Chest discomfort | 1.74 | 1.33-2.27 | <0.0001 |
| LDL-C level, mmol/L |  |  |  |
| LDL-C<1.81 | 1 | 1 |  |
| LDL-C:1.81-2.59 | 1.25 | 0.98-1.59 | 0.074 |
| LDL-C:2.6-3.37 | 1.49 | 1.16-1.90 | 0.002 |
| LDL-C> 3.37 | 1.68 | 1.18-2.37 | 0.004 |
| LDL-C Unrecorded | 0.68 | 0.49-0.94 | 0.020 |
| Urban/Rural |  |  |  |
| Urban | 1 | 1 |  |
| Rural | 0.52 | 0.27-1.00 | 0.051 |

Step 15

| Variables | Odds ratio | 95% Confidence interval | P value |
| --- | --- | --- | --- |
| Cardiovascular risk factors |  |  |  |
| Hypertension | 1.36 | 1.14-1.63 | 0.001 |
| Current smoker | 1.29 | 1.06-1.57 | 0.010 |
| Clinical characteristics at admission | |  |  |
| Chest discomfort | 1.76 | 1.34-2.30 | <0.0001 |
| LDL-C level, mmol/L |  |  |  |
| LDL-C<1.81 | 1 | 1 |  |
| LDL-C:1.81-2.59 | 1.26 | 0.99-1.61 | 0.063 |
| LDL-C:2.6-3.37 | 1.51 | 1.17-1.95 | 0.002 |
| LDL-C> 3.37 | 1.71 | 1.21-2.42 | 0.002 |
| LDL-C Unrecorded | 0.67 | 0.48-0.93 | 0.017 |
| Urban/Rural |  |  |  |
| Urban | 1 | 1 |  |
| Rural | 0.53 | 0.28-1.01 | 0.052 |

Step 16

| Variables | Odds ratio | 95% Confidence interval | P value |
| --- | --- | --- | --- |
| Cardiovascular risk factors |  |  |  |
| Hypertension | 1.44 | 1.19-1.75 | <0.0001 |
| Current smoker | 1.36 | 1.10-1.67 | 0.004 |
| Clinical characteristics at admission | |  |  |
| Chest discomfort | 1.75 | 1.34-2.29 | <0.0001 |
| LDL-C level, mmol/L |  |  |  |
| LDL-C<1.81 | 1 | 1 |  |
| LDL-C:1.81-2.59 | 1.25 | 0.99-1.57 | 0.064 |
| LDL-C:2.6-3.37 | 1.52 | 1.18-1.95 | 0.001 |
| LDL-C> 3.37 | 1.72 | 1.21-2.45 | 0.003 |
| LDL-C Unrecorded | 0.66 | 0.47-0.93 | 0.017 |
