# Supplementary material for: National Assessment of Statin Therapy in Patients Hospitalized with Acute Myocardial Infarction: Insight from China PEACE-Retrospective AMI Study, 2001, 2006, 2011
Source: PLoS One. 2016 Apr 8;11(4):e0150806. doi: 10.1371/journal.pone.0150806 (PMC4825974; doi:10.1371/journal.pone.0150806)
Supplement: S4 Appendix — (DOCX) [file pone.0150806.s004.docx]

**Procedures to Identify Factors Independently Associated with Intensive Statin Therapy in 2011**

Step 1

| Variables | Odds ratio | 95% Confidence interval | P value |
| --- | --- | --- | --- |
| Demographic |  |  |  |
| Age, years |  |  |  |
| Age<55 | 1 | 1 |  |
| Age 55-64 | 0.96 | 0.81-1.14 | 0.665 |
| Age 65-74 | 1.00 | 0.81-1.23 | 0.995 |
| Age≥75 | 1.01 | 0.78-1.30 | 0.960 |
| Gender |  |  |  |
| Male | 1 | 1 |  |
| Female | 0.80 | 0.69-0.93 | 0.003 |
| Cardiovascular risk factors |  |  |  |
| Hypertension | 1.06 | 0.91-1.23 | 0.437 |
| Diabetes | 1.02 | 0.88-1.18 | 0.756 |
| Dyslipidemia | 1.18 | 0.97-1.43 | 0.102 |
| Current smoker | 0.98 | 0.82-1.18 | 0.833 |
| Medical histories |  |  |  |
| Myocardial infarction | 1.43 | 1.13-1.79 | 0.023 |
| Ischemic stroke | 0.78 | 0.63-0.96 | 0.018 |
| Hemorrhagic stroke | 0.85 | 0.51-1.43 | 0.542 |
| Clinical characteristics at admission |  |  |  |
| Chest discomfort | 0.97 | 0.77-1.21 | 0.759 |
| Cardiac arrest | 0.84 | 0.56-1.25 | 0.391 |
| Cardiac shock | 1.23 | 0.93-1.63 | 0.138 |
| AMI type |  |  |  |
| NSTEMI | 1 | 1 |  |
| STEMI | 0.92 | 0.75-1.12 | 0.412 |
| In hospital management |  |  |  |
| PCI | 2.76 | 2.01-3.80 | <0.0001 |
| LDL-C level, mmol/L |  |  |  |
| LDL-C<1.81 | 1 | 1 |  |
| LDL-C:1.81-2.59 | 0.94 | 0.77-1.15 | 0.565 |
| LDL-C:2.6-3.37 | 0.98 | 0.78-1.23 | 0.844 |
| LDL-C> 3.37 | 1.00 | 0.74-1.36 | 0.979 |
| LDL-C Unrecorded | 0.68 | 0.47-0.99 | 0.047 |
| Region |  |  |  |
| Western | 1 | 1 |  |
| Eastern | 2.34 | 1.09-5.02 | 0.029 |
| Central | 1.34 | 0.60-3.01 | 0.480 |
| Urban/Rural |  |  |  |
| Urban | 1 | 1 |  |
| Rural | 0.37 | 0.22-0.63 | <0.0001 |

Step 2

| Variables | Odds ratio | 95% Confidence interval | P value |
| --- | --- | --- | --- |
| Demographic |  |  |  |
| Age, years |  |  |  |
| Age<55 | 1 | 1 |  |
| Age 55-64 | 0.96 | 0.81-1.15 | 0.681 |
| Age 65-74 | 1.00 | 0.82-1.23 | 0.979 |
| Age≥75 | 1.01 | 0.78-1.30 | 0.930 |
| Gender |  |  |  |
| Male | 1 | 1 |  |
| Female | 0.81 | 0.70-0.92 | 0.002 |
| Cardiovascular risk factors |  |  |  |
| Hypertension | 1.06 | 0.92-1.23 | 0.422 |
| Diabetes | 1.02 | 0.89-1.18 | 0.738 |
| Dyslipidemia | 1.18 | 0.97-1.43 | 0.103 |
| Medical histories |  |  |  |
| Myocardial infarction | 1.42 | 1.12-1.79 | 0.019 |
| Ischemic stroke | 0.78 | 0.63-0.96 | 0.018 |
| Hemorrhagic stroke | 0.85 | 0.51-1.43 | 0.546 |
| Clinical characteristics at admission | |  |  |
| Chest discomfort | 0.97 | 0.77-1.20 | 0.754 |
| Cardiac arrest | 0.84 | 0.56-1.25 | 0.391 |
| Cardiac shock | 1.23 | 0.93-1.63 | 0.139 |
| AMI type |  |  |  |
| NSTEMI | 1 | 1 |  |
| STEMI | 0.92 | 0.75-1.12 | 0.409 |
| In hospital management |  |  |  |
| PCI | 2.76 | 2.01-3.80 | <0.0001 |
| LDL-C level, mmol/L |  |  |  |
| LDL-C<1.81 | 1 | 1 |  |
| LDL-C:1.81-2.59 | 0.94 | 0.77-1.15 | 0.562 |
| LDL-C:2.6-3.37 | 0.98 | 0.78-1.23 | 0.840 |
| LDL-C> 3.37 | 1.00 | 0.74-1.36 | 0.981 |
| LDL-C Unrecorded | 0.68 | 0.47-1.00 | 0.047 |
| Region |  |  |  |
| Western | 1 | 1 |  |
| Eastern | 2.34 | 1.09-5.02 | 0.029 |
| Central | 1.34 | 0.60-3.01 | 0.480 |
| Urban/Rural |  |  |  |
| Urban | 1 | 1 |  |
| Rural | 0.37 | 0.22-0.63 | <0.0001 |

Step 3

| Variables | Odds ratio | 95% Confidence interval | P value |
| --- | --- | --- | --- |
| Demographic |  |  |  |
| Age, years |  |  |  |
| Age<55 | 1 | 1 |  |
| Age 55-64 | 0.96 | 0.81-1.15 | 0.683 |
| Age 65-74 | 1.00 | 0.82-1.23 | 0.973 |
| Age≥75 | 1.01 | 0.78-1.30 | 0.916 |
| Gender |  |  |  |
| Male | 1 | 1 |  |
| Female | 0.81 | 0.70-0.92 | 0.003 |
| Cardiovascular risk factors |  |  |  |
| Hypertension | 1.06 | 0.92-1.23 | 0.423 |
| Diabetes | 1.03 | 0.89-1.18 | 0.728 |
| Dyslipidemia | 1.18 | 0.97-1.43 | 0.103 |
| Medical histories |  |  |  |
| Myocardial infarction | 1.43 | 1.13-1.80 | 0.020 |
| Ischemic stroke | 0.78 | 0.63-0.96 | 0.019 |
| Hemorrhagic stroke | 0.85 | 0.51-1.43 | 0.549 |
| Clinical characteristics at admission | |  |  |
| Cardiac arrest | 0.84 | 0.56-1.26 | 0.400 |
| Cardiac shock | 1.23 | 0.94-1.63 | 0.137 |
| AMI type |  |  |  |
| NSTEMI | 1 | 1 |  |
| STEMI | 0.92 | 0.75-1.12 | 0.406 |
| In hospital management |  |  |  |
| PCI | 2.76 | 2.01-3.79 | <0.0001 |
| LDL-C level, mmol/L |  |  |  |
| LDL-C<1.81 | 1 | 1 |  |
| LDL-C:1.81-2.59 | 0.94 | 0.77-1.15 | 0.557 |
| LDL-C:2.6-3.37 | 0.98 | 0.78-1.23 | 0.832 |
| LDL-C> 3.37 | 1.00 | 0.74-1.36 | 0.987 |
| LDL-C Unrecorded | 0.68 | 0.47-1.00 | 0.047 |
| Region |  |  |  |
| Western | 1 | 1 |  |
| Eastern | 2.34 | 1.09-5.02 | 0.030 |
| Central | 1.34 | 0.59-3.01 | 0.482 |
| Urban/Rural |  |  |  |
| Urban | 1 | 1 |  |
| Rural | 0.37 | 0.22-0.63 | <0.0001 |

Step 4

| Variables | Odds ratio | 95% Confidence interval | | P value | |
| --- | --- | --- | --- | --- | --- |
| Demographic |  |  |  | |  |
| Age, years |  |  |  | |  |
| Age<55 | 1 | 1 |  | |  |
| Age 55-64 | 0.97 | 0.81-1.15 | 0.687 | |  |
| Age 65-74 | 1.00 | 0.82-1.23 | 0.971 | |  |
| Age≥75 | 1.01 | 0.78-1.31 | 0.920 | |  |
| Gender |  |  |  | |  |
| Male | 1 | 1 |  | |  |
| Female | 0.81 | 0.71-0.93 | 0.002 | |  |
| Cardiovascular risk factors |  |  |  | |  |
| Hypertension | 1.06 | 0.92-1.23 | 0.407 | |  |
| Dyslipidemia | 1.18 | 0.97-1.43 | 0.099 | |  |
| Medical histories |  |  |  | |  |
| Myocardial infarction | 1.44 | 1.12-1.79 | 0.023 | |  |
| Ischemic stroke | 0.78 | 0.63-0.96 | 0.019 | |  |
| Hemorrhagic stroke | 0.85 | 0.51-1.43 | 0.548 | |  |
| Clinical characteristics at admission | |  |  | |  |
| Cardiac arrest | 0.84 | 0.56-1.26 | 0.395 | |  |
| Cardiac shock | 1.23 | 0.94-1.63 | 0.136 | |  |
| AMI type |  |  |  | |  |
| NSTEMI | 1 | 1 |  | |  |
| STEMI | 0.92 | 0.75-1.12 | 0.399 | |  |
| In hospital management |  |  |  | |  |
| PCI | 2.76 | 2.01-3.79 | <0.0001 | |  |
| LDL-C level, mmol/L |  |  |  | |  |
| LDL-C<1.81 | 1 | 1 |  | |  |
| LDL-C:1.81-2.59 | 0.94 | 0.77-1.15 | 0.555 | |  |
| LDL-C:2.6-3.37 | 0.98 | 0.78-1.23 | 0.830 | |  |
| LDL-C> 3.37 | 1.00 | 0.74-1.36 | 0.992 | |  |
| LDL-C Unrecorded | 0.68 | 0.47-1.00 | 0.047 | |  |
| Region |  |  |  | |  |
| Western | 1 | 1 |  | |  |
| Eastern | 2.34 | 1.09-5.03 | 0.029 | |  |
| Central | 1.34 | 0.59-3.01 | 0.482 | |  |
| Urban/Rural |  |  |  | |  |
| Urban | 1 | 1 |  | |  |
| Rural | 0.37 | 0.22-0.63 | <0.0001 | |  |

Step 5

| Variables | Odds ratio | | | 95% Confidence interval | | | P value | |  |
| --- | --- | --- | --- | --- | --- | --- | --- | --- | --- |
| Demographic | |  | |  | |  | | |  |
| Gender | |  | |  | |  | | |  |
| Male | | 1 | | 1 | |  | | |  |
| Female | | 0.81 | | 0.71-0.93 | | 0.003 | | |  |
| Cardiovascular risk factors | |  | |  | |  | | |  |
| Hypertension | | 1.06 | | 0.92-1.23 | | 0.403 | | |  |
| Dyslipidemia | | 1.18 | | 0.97-1.43 | | 0.104 | | |  |
| Medical histories | |  | |  | |  | | |  |
| Myocardial infarction | | 1.43 | | 1.13-1.79 | | 0.022 | | |  |
| Ischemic stroke | | 0.78 | | 0.64-0.96 | | 0.020 | | |  |
| Hemorrhagic stroke | | 0.85 | | 0.51-1.42 | | 0.537 | | |  |
| Clinical characteristics at admission | |  | |  | |  | | |  |
| Cardiac arrest | | 0.84 | | 0.56-1.25 | | 0.392 | | |  |
| Cardiac shock | | 1.23 | | 0.94-1.63 | | 0.127 | | |  |
| AMI type | |  | |  | |  | | |  |
| NSTEMI | | 1 | | 1 | |  | | |  |
| STEMI | | | 0.92 | | 0.75-1.13 | | | 0.404 | |
| In hospital management | |  | |  | |  | | |  |
| PCI | | 2.74 | | 2.01-3.79 | | <0.0001 | | |  |
| LDL-C level, mmol/L | |  | |  | |  | | |  |
| LDL-C<1.81 | | 1 | | 1 | |  | | |  |
| LDL-C:1.81-2.59 | | 0.94 | | 0.77-1.15 | | 0.555 | | |  |
| LDL-C:2.6-3.37 | | 0.97 | | 0.77-1.22 | | 0.815 | | |  |
| LDL-C> 3.37 | | 1.00 | | 0.74-1.35 | | 0.999 | | |  |
| LDL-C Unrecorded | | 0.68 | | 0.47-1.00 | | 0.049 | | |  |
| Region | |  | |  | |  | | |  |
| Western | | 1 | | 1 | |  | | |  |
| Eastern | | 2.34 | | 1.09-5.03 | | 0.029 | | |  |
| Central | | 1.34 | | 0.59-3.01 | | 0.483 | | |  |
| Urban/Rural | |  | |  | |  | | |  |
| Urban | | 1 | | 1 | |  | | |  |
| Rural | | 0.37 | | 0.22-0.63 | | <0.0001 | | |  |

Step 6

| Variables | Odds ratio | | 95% Confidence interval | | | P value | |  |
| --- | --- | --- | --- | --- | --- | --- | --- | --- |
| Demographic |  | |  | |  | | |  |
| Gender |  | |  | |  | | |  |
| Male | 1 | | 1 | |  | | |  |
| Female | 0.81 | | 0.71-0.93 | | 0.004 | | |  |
| Cardiovascular risk factors |  | |  | |  | | |  |
| Hypertension | 1.06 | | 0.92-1.23 | | 0.422 | | |  |
| Dyslipidemia | 1.18 | | 0.97-1.43 | | 0.104 | | |  |
| Medical histories |  | |  | |  | | |  |
| Myocardial infarction | 1.42 | | 1.13-1.79 | | 0.023 | | |  |
| Ischemic stroke | 0.78 | | 0.64-0.96 | | 0.018 | | |  |
| Clinical characteristics at admission |  | |  | |  | | |  |
| Cardiac arrest | 0.84 | | 0.56-1.26 | | 0.396 | | |  |
| Cardiac shock | 1.24 | | 0.94-1.63 | | 0.126 | | |  |
| AMI type |  | |  | |  | | |  |
| NSTEMI | 1 | | 1 | |  | | |  |
| STEMI | | 0.91 | | 0.75-1.12 | | | 0.395 | |
| In hospital management |  | |  | |  | | |  |
| PCI | 2.74 | | 2.01-3.74 | | <0.0001 | | |  |
| LDL-C level, mmol/L |  | |  | |  | | |  |
| LDL-C<1.81 | 1 | | 1 | |  | | |  |
| LDL-C:1.81-2.59 | 0.94 | | 0.77-1.15 | | 0.556 | | |  |
| LDL-C:2.6-3.37 | 0.97 | | 0.77-1.22 | | 0.812 | | |  |
| LDL-C> 3.37 | 1.00 | | 0.74-1.35 | | 0.999 | | |  |
| LDL-C Unrecorded | 0.68 | | 0.47-1.00 | | 0.048 | | |  |
| Region |  | |  | |  | | |  |
| Western | 1 | | 1 | |  | | |  |
| Eastern | 2.34 | | 1.09-5.03 | | 0.029 | | |  |
| Central | 1.34 | | 0.59-3.01 | | 0.483 | | |  |
| Urban/Rural |  | |  | |  | | |  |
| Urban | 1 | | 1 | |  | | |  |
| Rural | 0.37 | | 0.22-0.63 | | <0.0001 | | |  |

Step 7

| Variables | Odds ratio | | | 95% Confidence interval | | | P value | |  |  |
| --- | --- | --- | --- | --- | --- | --- | --- | --- | --- | --- |
| Demographic | |  | |  | | |  | |  |  |
| Gender | |  | |  | |  | | | |  |
| Male | | 1 | | 1 | |  | | | |  |
| Female | | 0.81 | | 0.71-0.93 | | 0.003 | | | |  |
| Cardiovascular risk factors | |  | |  | |  | | | |  |
| Hypertension | | 1.06 | | 0.92-1.23 | | 0.409 | | | |  |
| Dyslipidemia | | 1.18 | | 0.97-1.43 | | 0.101 | | | |  |
| Medical histories | |  | |  | |  | | | |  |
| Myocardial infarction | | 1.43 | | 1.13-1.79 | | 0.021 | | | |  |
| Ischemic stroke | | 0.78 | | 0.64-0.96 | | 0.019 | | | |  |
| Clinical characteristics at admission | |  | |  | |  | | | |  |
| Cardiac arrest | | 0.84 | | 0.56-1.26 | | 0.398 | | | |  |
| Cardiac shock | | 1.24 | | 0.94-1.63 | | 0.125 | | | |  |
| AMI type | |  | |  | |  | | | |  |
| NSTEMI | | 1 | | 1 | |  | | | |  |
| STEMI | | | 0.91 | | 0.74-1.12 | | | 0.391 | | |
| In hospital management | |  | |  | |  | | | |  |
| PCI | | 2.74 | | 2.01-3.74 | | <0.0001 | | | |  |
| LDL-C level, mmol/L | |  | |  | |  | | | |  |
| LDL-C<1.81 | | 1 | | 1 | |  | | | |  |
| LDL-C:1.81-2.59 | | 0.94 | | 0.77-1.15 | | 0.549 | | | |  |
| LDL-C:2.6-3.37 | | 0.97 | | 0.77-1.22 | | 0.797 | | | |  |
| LDL-C> 3.37 | | 1.00 | | 0.74-1.35 | | 0.988 | | | |  |
| LDL-C Unrecorded | | 0.68 | | 0.47-1.00 | | 0.048 | | | |  |
| Region | |  | |  | |  | | | |  |
| Western | | 1 | | 1 | |  | | | |  |
| Eastern | | 2.35 | | 1.09-5.05 | | 0.029 | | | |  |
| Central | | 1.34 | | 0.60-3.01 | | 0.481 | | | |  |
| Urban/Rural | |  | |  | |  | | | |  |
| Urban | | 1 | | 1 | |  | | | |  |
| Rural | | 0.37 | | 0.22-0.63 | | <0.0001 | | | |  |

Step 8

| Variables | Odds ratio | | | 95% Confidence interval | | P value | |  |
| --- | --- | --- | --- | --- | --- | --- | --- | --- |
| Demographic | |  | |  | |  | |  |
| Gender | |  | |  | |  | |  |
| Male | | 1 | | 1 | |  | |  |
| Female | | 0.81 | | 0.71-0.93 | | 0.003 | |  |
| Cardiovascular risk factors | |  | |  | |  | |  |
| Hypertension | | 1.06 | | 0.92-1.23 | | 0.420 | |  |
| Dyslipidemia | | 1.17 | | 0.96-1.43 | | 0.112 | |  |
| Medical histories | |  | |  | |  | |  |
| Myocardial infarction | | 1.43 | | 1.13-1.79 | | 0.023 | |  |
| Ischemic stroke | | 0.78 | | 0.64-0.96 | | 0.018 | |  |
| Clinical characteristics at admission | |  | |  | |  | |  |
| Cardiac arrest | | 0.86 | | 0.57-1.28 | | 0.449 | |  |
| Cardiac shock | | 1.25 | | 0.94-1.64 | | 0.099 | |  |
| AMI type | |  | |  | |  | |  |
| NSTEMI | | 1 | | 1 | |  | |  |
| STEMI | | | 0.93 | | 0.75-1.15 | | 0.501 | |
| In hospital management | |  | |  | |  | |  |
| PCI | | 2.74 | | 2.01-3.74 | | <0.0001 | |  |
| LDL-C level, mmol/L | |  | |  | |  | |  |
| LDL-C<1.81 | | 1 | | 1 | |  | |  |
| LDL-C:1.81-2.59 | | 0.94 | | 0.77-1.16 | | 0.573 | |  |
| LDL-C:2.6-3.37 | | 0.98 | | 0.78-1.23 | | 0.854 | |  |
| LDL-C> 3.37 | | 1.01 | | 0.75-1.36 | | 0.957 | |  |
| LDL-C Unrecorded | | 0.67 | | 0.46-0.98 | | 0.039 | |  |
| Region | |  | |  | |  | |  |
| Western | | 1 | | 1 | |  | |  |
| Eastern | | 2.35 | | 1.09-5.07 | | 0.029 | |  |
| Central | | 1.34 | | 0.60-3.02 | | 0.478 | |  |
| Urban/Rural | |  | |  | |  | |  |
| Urban | | 1 | | 1 | |  | |  |
| Rural | | 0.37 | | 0.22-0.64 | | <0.0001 | |  |

Step 9

| Variables | Odds ratio | | 95% Confidence interval | | P value |
| --- | --- | --- | --- | --- | --- |
| Demographic | |  | |  |  |
| Gender | |  | |  |  |
| Male | | 1 | | 1 |  |
| Female | | 0.81 | | 0.70-0.93 | 0.004 |
| Cardiovascular risk factors | |  | |  |  |
| Hypertension | | 1.07 | | 0.92-1.24 | 0.385 |
| Dyslipidemia | | 1.17 | | 0.96-1.43 | 0.111 |
| Medical histories | |  | |  |  |
| Myocardial infarction | | 1.42 | | 1.12-1.78 | 0.023 |
| Ischemic stroke | | 0.78 | | 0.64-0.96 | 0.019 |
| Clinical characteristics at admission | |  | |  |  |
| Cardiac arrest | | 0.85 | | 0.57-1.27 | 0.433 |
| Cardiac shock | | 1.24 | | 0.94-1.63 | 0.116 |
| In hospital management | |  | |  |  |
| PCI | | 2.73 | | 2.01-3.70 | <0.0001 |
| LDL-C level, mmol/L | |  | |  |  |
| LDL-C<1.81 | | 1 | | 1 |  |
| LDL-C:1.81-2.59 | | 0.94 | | 0.77-1.16 | 0.566 |
| LDL-C:2.6-3.37 | | 0.98 | | 0.78-1.23 | 0.844 |
| LDL-C> 3.37 | | 1.01 | | 0.75-1.36 | 0.961 |
| LDL-C Unrecorded | | 0.67 | | 0.46-0.98 | 0.040 |
| Region | |  | |  |  |
| Western | | 1 | | 1 |  |
| Eastern | | 2.36 | | 1.09-5.08 | 0.029 |
| Central | | 1.34 | | 0.60-3.02 | 0.477 |
| Urban/Rural | |  | |  |  |
| Urban | | 1 | | 1 |  |
| Rural | | 0.37 | | 0.22-0.64 | <0.0001 |

Step 10

| Variables | Odds ratio | 95% Confidence interval | P value |
| --- | --- | --- | --- |
| Demographic |  |  |  |
| Gender |  |  |  |
| Male | 1 | 1 |  |
| Female | 0.81 | 0.71-0.94 | 0.004 |
| Cardiovascular risk factors |  |  |  |
| Hypertension | 1.07 | 0.92-1.24 | 0.384 |
| Dyslipidemia | 1.17 | 0.96-1.43 | 0.112 |
| Medical histories |  |  |  |
| Myocardial infarction | 1.42 | 1.12-1.78 | 0.022 |
| Ischemic stroke | 0.78 | 0.64-0.96 | 0.019 |
| Clinical characteristics at admission |  |  |  |
| Cardiac shock | 1.23 | 0.94-1.61 | 0.128 |
| In hospital management |  |  |  |
| PCI | 2.72 | 2.01-3.69 | <0.0001 |
| LDL-C level, mmol/L |  |  |  |
| LDL-C<1.81 | 1 | 1 |  |
| LDL-C:1.81-2.59 | 0.94 | 0.77-1.16 | 0.574 |
| LDL-C:2.6-3.37 | 0.98 | 0.78-1.23 | 0.855 |
| LDL-C> 3.37 | 1.01 | 0.75-1.36 | 0.953 |
| LDL-C Unrecorded | 0.67 | 0.46-0.98 | 0.040 |
| Region |  |  |  |
| Western | 1 | 1 |  |
| Eastern | 2.35 | 1.09-5.07 | 0.029 |
| Central | 1.34 | 0.60-3.02 | 0.477 |
| Urban/Rural |  |  |  |
| Urban | 1 | 1 |  |
| Rural | 0.37 | 0.22-0.64 | <0.0001 |

Step 11

| Variables | Odds ratio | | 95% Confidence interval | | | P value | |
| --- | --- | --- | --- | --- | --- | --- | --- |
| Demographic | |  | |  |  | |  |
| Gender | |  | |  |  | |  |
| Male | | 1 | | 1 |  | |  |
| Female | | 0.82 | | 0.71-0.94 | 0.006 | |  |
| Cardiovascular risk factors | |  | |  |  | |  |
| Dyslipidemia | | 1.18 | | 0.97-1.43 | 0.105 | |  |
| Medical histories | |  | |  |  | |  |
| Myocardial infarction | | 1.43 | | 1.12-1.80 | 0.023 | |  |
| Ischemic stroke | | 0.79 | | 0.64-0.98 | 0.028 | |  |
| Clinical characteristics at admission | |  | |  |  | |  |
| Cardiac shock | | 1.23 | | 0.94-1.60 | 0.135 | |  |
| In hospital management | |  | |  |  | |  |
| PCI | | 2.72 | | 2.01-3.69 | <0.0001 | |  |
| LDL-C level, mmol/L | |  | |  |  | |  |
| LDL-C<1.81 | | 1 | | 1 |  | |  |
| LDL-C:1.81-2.59 | | 0.94 | | 0.77-1.16 | 0.563 | |  |
| LDL-C:2.6-3.37 | | 0.98 | | 0.78-1.23 | 0.851 | |  |
| LDL-C> 3.37 | | 1.01 | | 0.75-1.36 | 0.965 | |  |
| LDL-C Unrecorded | | 0.67 | | 0.46-0.98 | 0.041 | |  |
| Region | |  | |  |  | |  |
| Western | | 1 | | 1 |  | |  |
| Eastern | | 2.36 | | 1.10-5.09 | 0.028 | |  |
| Central | | 1.34 | | 0.60-3.02 | 0.477 | |  |
| Urban/Rural | |  | |  |  | |  |
| Urban | | 1 | | 1 |  | |  |
| Rural | | 0.37 | | 0.22-0.63 | <0.0001 | |  |

Step 12

| Variables | Odds ratio | 95% Confidence interval | | P value |
| --- | --- | --- | --- | --- |
| Demographic |  | |  |  |
| Gender |  | |  |  |
| Male | 1 | | 1 |  |
| Female | 0.82 | | 0.71-0.95 | 0.007 |
| Cardiovascular risk factors |  | |  |  |
| Dyslipidemia | 1.18 | | 0.96-1.43 | 0.108 |
| Medical histories |  | |  |  |
| Myocardial infarction | 1.43 | | 1.12-1.80 | 0.023 |
| Ischemic stroke | 0.79 | | 0.64-0.98 | 0.029 |
| In hospital management |  | |  |  |
| PCI | 2.72 | | 2.00-3.68 | <0.0001 |
| LDL-C level, mmol/L |  | |  |  |
| LDL-C<1.81 | 1 | | 1 |  |
| LDL-C:1.81-2.59 | 0.94 | | 0.76-1.15 | 0.526 |
| LDL-C:2.6-3.37 | 0.97 | | 0.78-1.22 | 0.805 |
| LDL-C> 3.37 | 1.00 | | 0.74-1.35 | 0.997 |
| LDL-C Unrecorded | 0.67 | | 0.46-0.98 | 0.037 |
| Region |  | |  |  |
| Western | 1 | | 1 |  |
| Eastern | 2.36 | | 1.10-5.09 | 0.028 |
| Central | 1.34 | | 0.60-3.02 | 0.477 |
| Urban/Rural |  | |  |  |
| Urban | 1 | | 1 |  |
| Rural | 0.37 | | 0.22-0.63 | <0.001 |

Step 13

| Variables | Odds ratio | 95% Confidence interval | P value |
| --- | --- | --- | --- |
| Demographic |  |  |  |
| Gender |  |  |  |
| Male | 1 | 1 |  |
| Female | 0.85 | 0.74-0.98 | 0.012 |
| Medical histories |  |  |  |
| Myocardial infarction | 1.42 | 1.13-1.79 | 0.022 |
| Ischemic stroke | 0.80 | 0.65-0.98 | 0.030 |
| In hospital management |  |  |  |
| PCI | 2.70 | 1.99-3.66 | <0.001 |
| LDL-C level, mmol/L |  |  |  |
| LDL-C<1.81 | 1 | 1 |  |
| LDL-C:1.81-2.59 | 0.92 | 0.75-1.13 | 0.492 |
| LDL-C:2.6-3.37 | 0.97 | 0.77-1.22 | 0.858 |
| LDL-C> 3.37 | 1.04 | 0.79-1.39 | 0.725 |
| LDL-C Unrecorded | 0.61 | 0.43-0.86 | 0.006 |
| Region |  |  |  |
| Western | 1 | 1 |  |
| Eastern | 2.39 | 1.11-5.12 | 0.027 |
| Central | 1.35 | 0.60-3.04 | 0.480 |
| Urban/Rural |  |  |  |
| Urban | 1 | 1 |  |
| Rural | 0.37 | 0.21-0.63 | <0.0001 |
