# Supplementary figures and images for: National Assessment of Statin Therapy in Patients Hospitalized with Acute Myocardial Infarction: Insight from China PEACE-Retrospective AMI Study, 2001, 2006, 2011
Source: PLoS One. 2016 Apr 8;11(4):e0150806. doi: 10.1371/journal.pone.0150806 (PMC4825974; doi:10.1371/journal.pone.0150806)

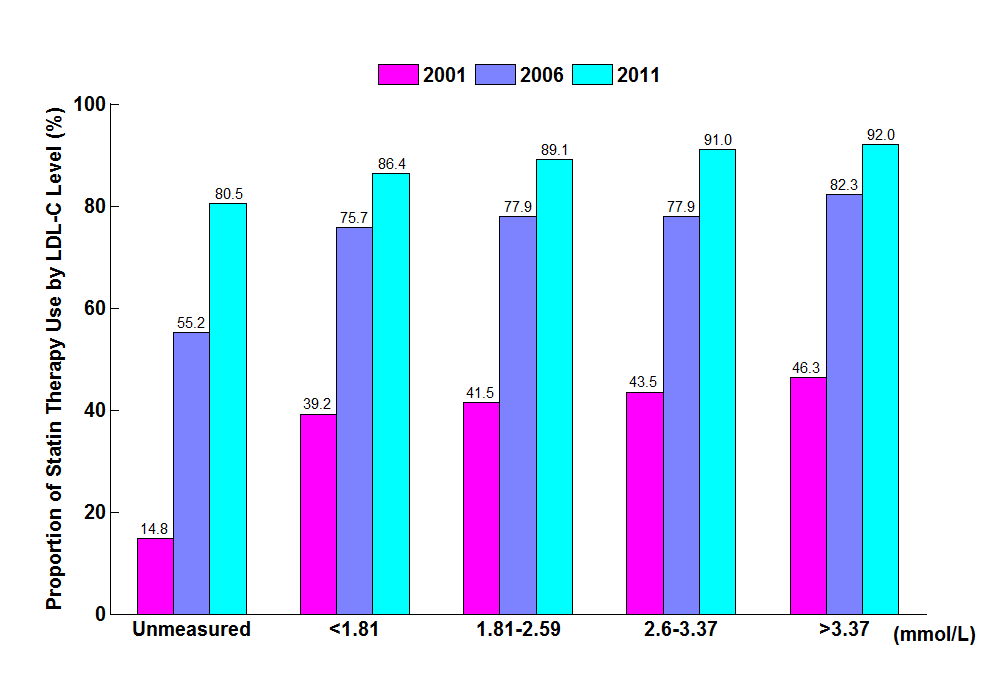

Supplement: S1 Fig — (TIF) [file pone.0150806.s005.tif]

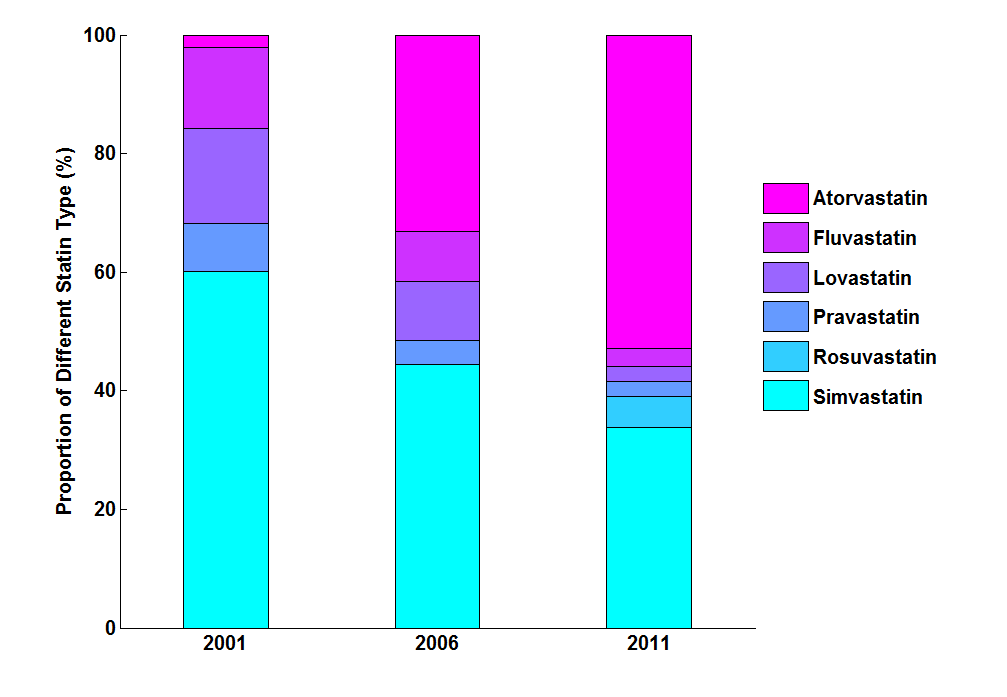

Supplement: S2 Fig — (TIF) [file pone.0150806.s006.tif]
