# Supplementary material for: National Assessment of Statin Therapy in Patients Hospitalized with Acute Myocardial Infarction: Insight from China PEACE-Retrospective AMI Study, 2001, 2006, 2011
Source: PLoS One. 2016 Apr 8;11(4):e0150806. doi: 10.1371/journal.pone.0150806 (PMC4825974; doi:10.1371/journal.pone.0150806)
Supplement: S1 Table — (DOCX) [file pone.0150806.s007.docx]

**S1 Table : Definition of statin equivalency**^10,20^ **(intensive statin therapy highlighted)**

|  | **Atorvastatin** | **Fluvastatin** | **Lovastatin** | **Pravastatin** | **Rosuvastatin** | **Simvastatin** | **Pitavastatin** |
| --- | --- | --- | --- | --- | --- | --- | --- |
| **Approved in China** | Yes | Yes | Yes | Yes | Yes | Yes | Yes |
| **Year of generic** | 1999 | 2007 | 1996 | 2005 | Not available | 1996 | 2009 |
| **%LDL-C Reduction** |  |  |  |  |  |  |  |
| 10-20% | _ | ≤20mg | ≤10mg | ≤10mg | _ | ≤5mg | - |
| 20-30% | _ | 40mg | 20mg | 20mg | _ | 10mg | - |
| 30-40% | ≤10mg | 80mg | 40mg | 40mg | ≤5mg | 20mg | 2mg |
| **40-50%** | **20-40mg** | **_** | **80mg** | **80mg** | **10mg** | **40-80mg** | **4mg** |
| **50-60%** | **80mg** | **_** | **_** | **_** | **20-40mg** | **_** |  |
